# One‐Pot Chemoenzymatic Synthesis of Microviridin Analogs Containing Functional Tags

**DOI:** 10.1002/cbic.202200345

**Published:** 2022-09-13

**Authors:** Stella Scholz, Sofia Kerestetzopoulou, Vincent Wiebach, Romina Schnegotzki, Bianca Schmid, Emmanuel Reyna‐González, Ling Ding, Roderich D. Süssmuth, Elke Dittmann, Martin Baunach

**Affiliations:** ^1^ Department of Microbiology University of Potsdam Karl-Liebknecht-Str. 24/25 14476 Potsdam-Golm Germany; ^2^ Institute of Chemistry Technical University Berlin Straße des 17. Juni 124 10623 Berlin Germany; ^3^ Department of Biotechnology and Biomedicine Technical University of Denmark Søltofts Plads, Building 221 DK-2800 Kgs. Lyngby Denmark; ^4^ Institute of Pharmaceutical Biology University of Bonn Nussallee 6 53115 Bonn Germany

**Keywords:** chemoenzymatic synthesis, protease inhibitors, microviridins, RiPPs, synthetic biology

## Abstract

Microviridins are a prominent family of ribosomally synthesized and posttranslationally modified peptides (RiPPs) featuring characteristic lactone and lactam rings. Their unusual cage‐like architecture renders them highly potent serine protease inhibitors of which individual variants specifically inhibit different types of proteases of pharmacological interest. While posttranslational modifications are key for the stability and bioactivity of RiPPs, additional attractive properties can be introduced by functional tags. To date – although highly desirable – no method has been reported to incorporate functional tags in microviridin scaffolds or the overarching class of graspetides. In this study, a chemoenzymatic *in vitro* platform is used to introduce functional tags in various microviridin variants yielding biotinylated, dansylated or propargylated congeners. This straightforward approach paves the way for customized protease inhibitors with built‐in functionalities that can help to unravel the still elusive ecological roles and targets of this remarkable class of compounds and to foster applications based on protease inhibition.

## Graphical abstract

**Tag it easy**: A chemoenzymatic strategy enabled the synthesis of microviridins with various functional tags from synthetic core peptides. This versatile and straightforward approach opens the toolbox of microviridin chemical probes to explore the still elusive ecological functions and targets of this remarkable class of compounds and to advance applications based on protease inhibition.

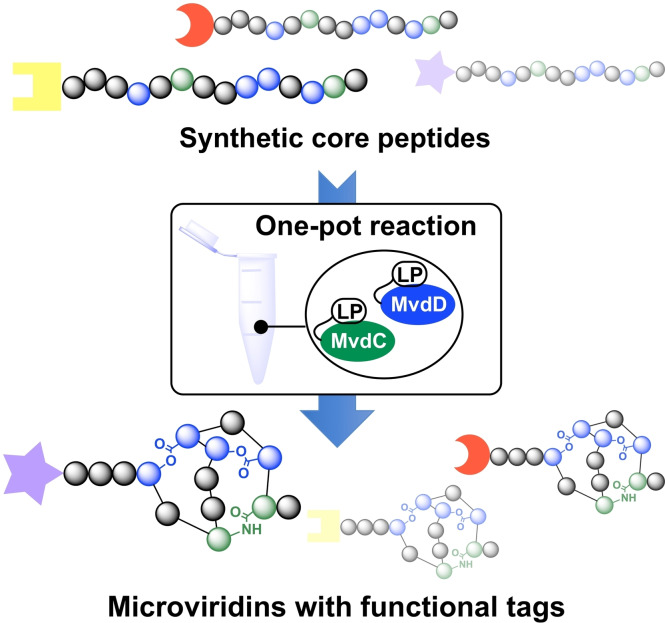

## Introduction

**Figure 1:**
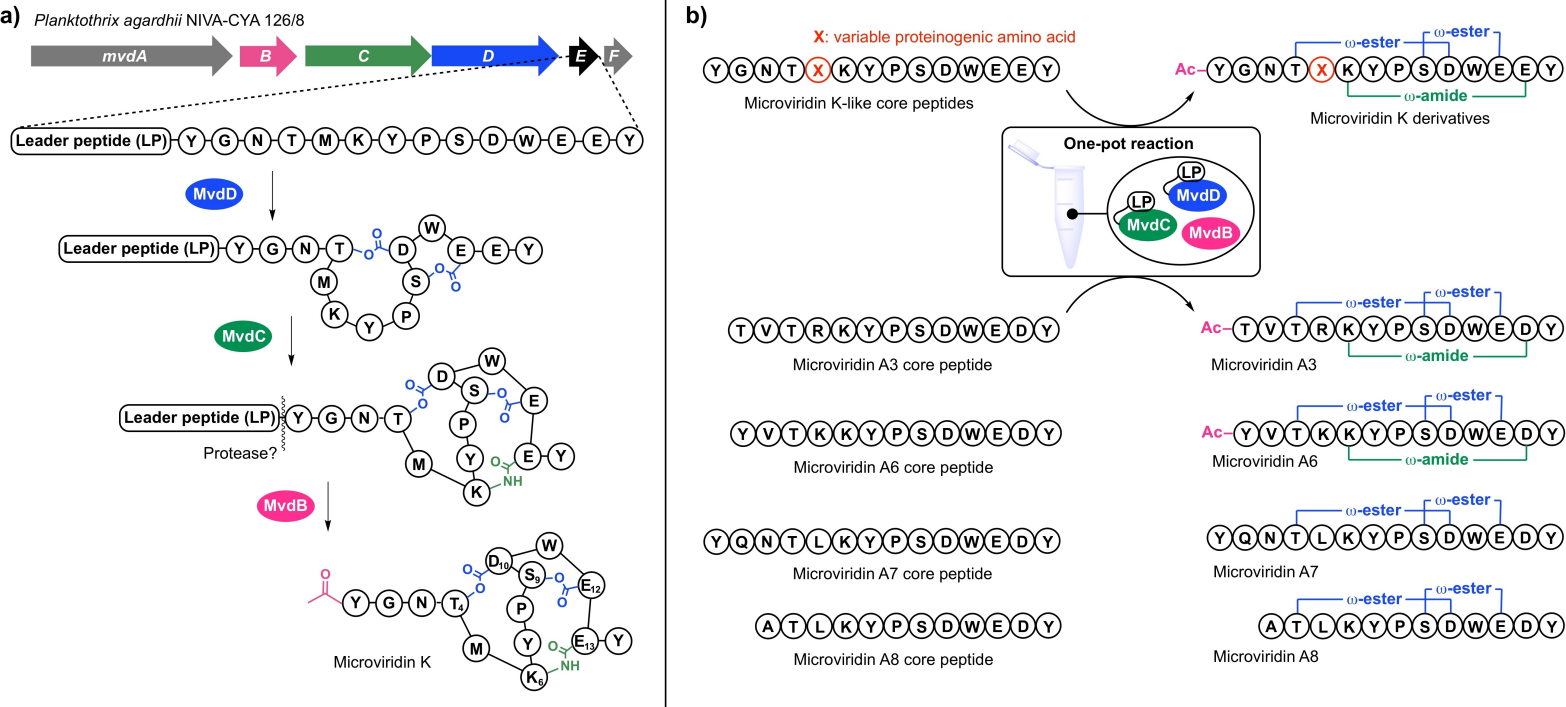
(Bio)synthesis of microviridin congeners. a) Biosynthesis of microviridin K in the strain *Planktothrix agardhii* NIVA‐CYA 126/8 comprising the lactone and lactam forming ATP‐grasp ligases MvdD and MvdC and the acetyltransferase MvdB.^[1]^ b) Chemoenzymatic *in vitro* synthesis platform: Generation of a microviridin K variant library by transforming synthetic core peptides into mature microviridin scaffolds in a one‐pot reaction with the help of leader peptide(LP)‐fused ATP‐grasp ligases LP‐MvdD and LP‐MvdC^[3]^ as well as transformation of synthetic core peptides of cryptic microviridin precursor peptides identified by genome mining.^[4]^

Microviridins are a prominent family of ribosomally synthesized and post‐translationally modified peptides (RiPPs).^[2]^ Due to strong inhibitory activity against serine proteases such as trypsin, chymotrypsin, subtilisin, and elastase they have been recognized for their potential use in the treatment of pathological conditions like pulmonary emphysema.^[2b]^ The hallmark of this compound class is their characteristic cage‐like architecture comprising two lactone linkages between the side chain carboxy group of Asp in position 10 of the core peptide (Asp_10_) with the hydroxy group of Thr_4_ and the side chain carboxy group of Glu_12_ with hydroxy group of Ser_9_, and a lactam linkage between the δ‐carboxy group of Glu/Asp_13_ with the ϵ‐amino group of Lys_6_ (Figure 1a).^[2]^ The lactones and lactams are sequentially installed on the precursor peptide by two dedicated ATP‐grasp ligases, MvdD and MvdC (alternatively referred to as MdnC and MdnB),^[5]^ followed by leader peptide cleavage and the subsequent acetylation at the N‐terminus by a *N*‐acetyltransferase.[^1^, ^2b^] Recently, related ω‐ester containing peptides with different ring topologies like plesiocin, thuringinin, and thatisin were discovered, which share lactone ring formation catalyzed by homologous ATP‐grasp ligases but lack a lactam ring.^[6]^ Together with the microviridins they form the newly introduced class of ‘graspetides’.[^2b^, ^7^]

While the bioactivity and mode of action against mammalian serine proteases is well investigated for microviridins[^2a^, ^8^] and plesiocin,^[9]^ their ecological roles and native targets remain elusive. Pioneering studies, which demonstrated microviridin J to cause lethal molting disruption in microcrustaceans of the genus *Daphnia* that feed on planktonic cyanobacteria point to a role as grazing deterrents.^[10]^ However, neither specific protease targets could be identified, nor could a mode of action be specified. One hypothesis is based on the depletion of the amino acid pool essential for production of a new integument due to incomplete protein digestion after the uptake of microviridin J. Another hypothesis is in favor of the direct inhibition of trypsin‐like proteases that are involved in the molting process itself.^[10b]^ In addition, little is known about the intra‐ and extracellular distribution of these potent toxins. While microviridin biosynthesis gene clusters frequently encode ABC transporters that were suggested to facilitate microviridin export,^[4]^ analytical studies imply a predominant localization of microviridins within the cells and only a scarce extracellular secretion.^[11]^ Thus, microviridins may play additional roles as intrinsic factors cyanobacterial ecology, like recently shown for the peptidic cyanotoxin microcystin,^[12]^ that have yet to be discovered. To fill these knowledge gaps, effective tools are urgently needed to monitor the intracellular and extracellular distribution of microviridins and to track the protein targets of microviridin ligands. In addition, 23 further groups of graspetides have recently been discovered or predicted, all of which still await functional characterization.^[6d]^ As an initial step, we planned to develop microviridin chemical probes with functional groups to utilize in biological assays. While affinity tags can be used for the identification of interaction partners in pull‐down experiments, fluorophores could be used to monitor the binding of targets. In addition, probes with clickable chemical handles that can be customized later on would be highly desirable, not only to have the opportunity to couple a diverse set of probes for bio‐orthogonal applications via copper‐catalyzed alkyne‐azide cycloadditions (CuAAC or click‐chemistry), but also to use microviridins in applications such as functionalized surfaces or drug conjugates.

Based on our long‐standing interest in the biosynthesis and engineering of microviridins,[^3^, ^4^, ^5^, ^8^, ^13^] we decided to use enzyme catalysis in synthesis trails to generate the envisioned chemical probes. Previously, we have efficiently established the *in vitro* reconstitution of microviridins based on two ATP‐grasp ligases from the biosynthesis pathway of microviridin K (Figure 1a) that are constitutively activated using a covalently attached leader peptide and a GNAT‐type *N*‐acetyltransferase.^[3]^ Constitutive activation was achieved by fusion of the MvdE leader peptide (LP) of *Planktothrix agardhii* NIVA‐CYA 126/8 with the N‐termini of the two ATP‐grasp ligases MvdD and MvdC, joined by a 30 amino acid linker, yielding LP‐MvdD and LP‐MvdC. This platform could be used for the efficient one‐pot transformation of microviridin core peptides to mature microviridins, as shown for the generation of microviridin variant libraries to screen and optimize protease inhibitors,^[3]^ as well as in genome mining approaches to bring cryptic microviridins to life (Figure 1b).^[4]^

Here we show that the chemoenzymatic technology could be used to introduce a variety of functional tags with attractive properties in non‐native microviridin core peptides, owing to the remarkable substrate tolerance of the constitutively activated ATP‐grasp ligases. Thus we could generate a versatile set of microviridin chemical probes employing *N* ^*ϵ*^‐biotinyl‐l‐lysine as an affinity tag, a dansyl moiety as a fluorophore or *O*‐propargyl‐l‐tyrosine as a clickable handle. Notably, the modified compounds retained full or at least partial activity against trypsin and elastase, respectively, allowing their immediate use as molecular tools. As a proof of concept, we used dansylated microviridin B as a diagnostic tool to selectively label elastase in protease mixtures.

## Results and Discussion

The core peptide of microviridin J (MvJ) was chosen for library construction due to its proposed role as a grazing deterrent in the interaction of *Microcystis* with microcrustaceans.^[10]^ Microviridin J chemical probes may thus help to identify the compound's natural targets and to elucidate the molecular basis of this intriguing antibiosis. In addition, microviridin B (MvB) was chosen as a second target, which – as a potent elastase inhibitor^[14]^ – functionally complements the trypsin inhibitor microviridin J. Peptides were chemically synthesized by solid‐phase‐peptide synthesis (SPPS, synthetic protocols see Supporting Information). To generate core peptides (CP) with an affinity tag *N* ^*ϵ*^‐biotinyl‐l‐lysine was introduced either at the N‐ or C‐terminus of the peptide, yielding MvJ_CP_N(Bio), MvJ_CP_C(Bio), MvB_CP_N(Bio), and MvB_CP_C(Bio). To generate core peptides with a clickable handle *O*‐propargyl‐l‐tyrosine was introduced either at the N‐ or C‐terminus of MvJ_CB, yielding MvJ_CP_N(Prop) and MvJ_CP_C(Prop). To generate a core peptide with a fluorophore a dansyl group was introduced at the N‐terminus of MvB_CP yielding MvB_CP_N(dansyl). Finally, MvJ_CP and MvB_CP were synthesized to serve as positive controls.

**Figure 2:**
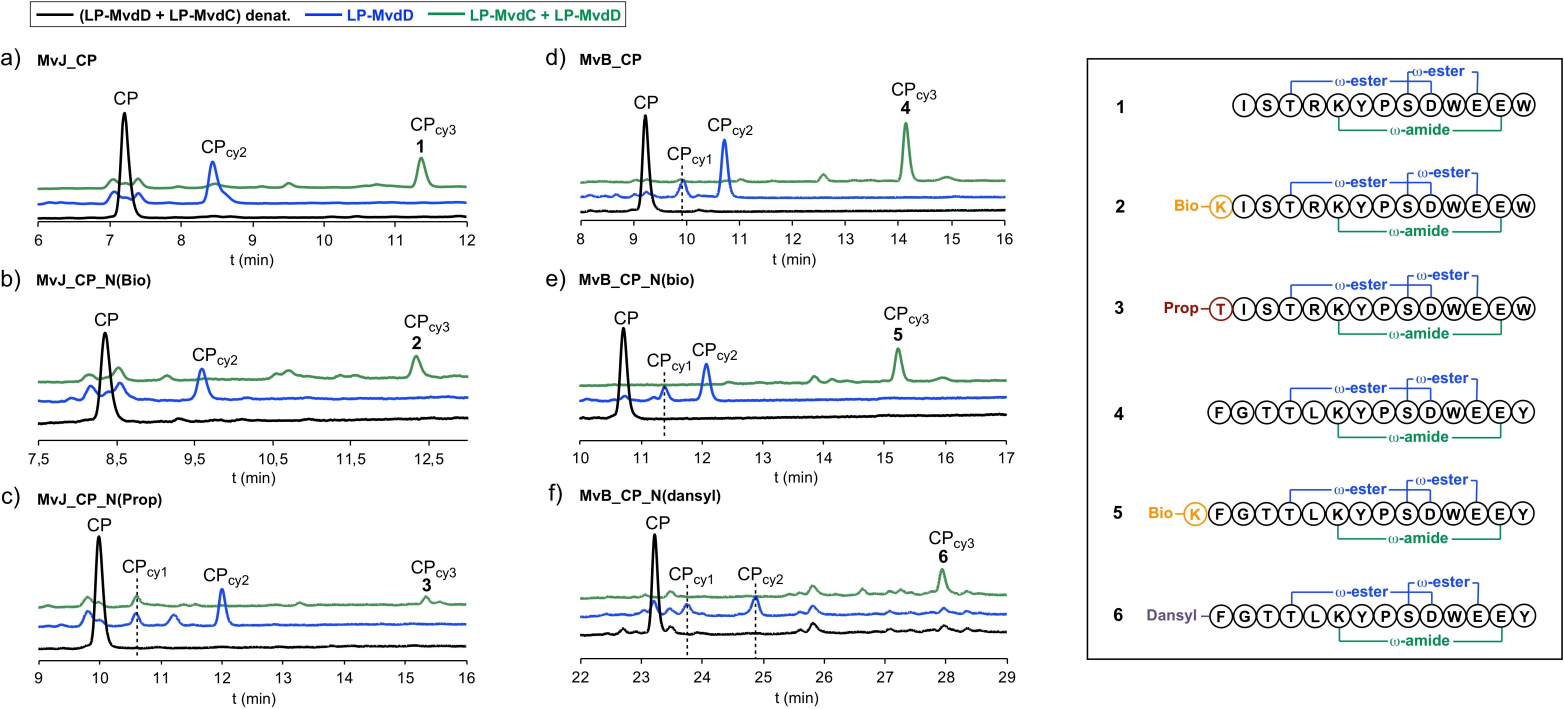
Chemoenzymatic synthesis of N‐terminally modified microviridin J and microviridin B derivatives by using LP‐MvdD alone (blue line) or in combination with LP‐MvdC (green line). a)–f) HPLC monitoring (absorbance at 199 nm) of enzyme assays. Novel peaks correspond to monocyclic (CP_cy1_), bicyclic (CP_cy2_) and tricyclic (CP_cy3_) microviridin products as indicated by mass spectrometric analysis (Figures S2–S29, Table S1). All tricyclic microviridins (**1**–**6**) were characterized and confirmed by tandem ESI‐MS/MS experiments (Figures S30–S35).

**Figure 3:**
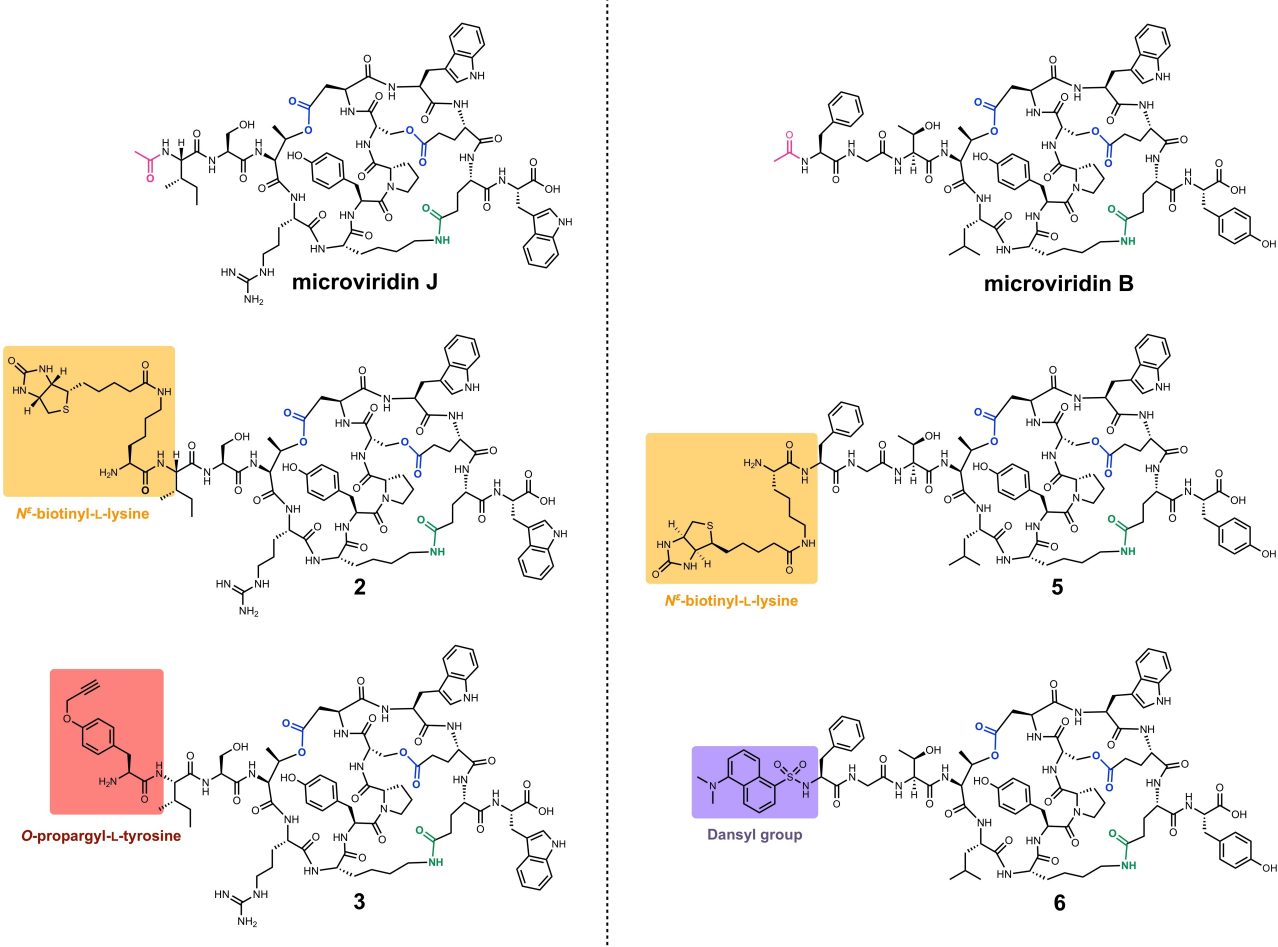
Structures of microviridin J and microviridin B together with microviridin J‐ and microviridin B‐type chemical probes employing either the affinity tag *N* ^*ϵ*^‐biotinyl‐l‐lysine (**2**; **5**), *O*‐propargyl‐l‐tyrosine as a clickable handle (**3**) or a dansyl moiety as a fluorophore (**6**).

Next, all modified and unmodified core peptides were subjected to *in vitro* enzyme assays with either the recombinant lactone forming ATP‐grasp ligase LP‐MvdD alone or with LP‐MvdD in combination with the lactam forming ATP‐grasp ligase LP‐MvdC. HPLC monitoring of the enzyme assays indicated, that all core peptides could be converted by LP‐MvdD due to the emergence of new peaks in combination with core peptide consumption (Figure 2, Figure S1). Mass spectrometric analysis indicated, that the new peaks correspond to monocyclic (CP_cy1_) or bicyclic (CP_cy2_) microviridin products as suggested by mass shifts of −18 Da and −36 Da (Figure S3–30; Table S1). These mass shifts could be explained by the loss of one or two water molecules, which is a consequence of the lactone ring formations. Remarkably, all N‐terminally modified core peptides could be converted to the bicyclic products (Figure 2), whereas the transformation apparently stopped after monocyclization for all C‐terminally modified core peptides (Figure S1). In addition, all N‐terminally modified core peptides – alike the positive controls – could be fully converted to tricyclic microviridins (CP_cy3_) (Figure 2; Table S1), thereby highlighting the versatility of the *in vitro* platform. These results suggest that the system might be flexibly extendable to further modification at the N‐terminal end. In contrast to that, the monocyclic C‐terminal modified variants could not be cyclized further by addition of LP‐MvdC (Figure S1), which is in line with the observation that double lactonization is a requirement for lactamization.^[15]^ The structures of all tricyclic microviridins (Figure 3) were characterized and confirmed by tandem ESI‐MS/MS experiments (Figures S31–S36). For the N‐terminally modified core peptides it seems that the efficiency of the chemoenzymatic conversion is more or less comparable to the unmodified core peptides, except for MvJ_CP_N(Prop), for which much smaller amounts of tricyclic product could be detected (Figure 2). This result is supported by a duplicate of the conversion experiment (Figure S2) as well as by the relatively poor overall percent yield of the enzymatic conversion of only 6 %, compared to the overall percent yields of the other substrates, which range from 19 %–26 % (Table S2).

The introduction of functional tags in RiPP natural products to further improve their complexity and endow them with attractive properties is a highly desired goal in peptide engineering.^[2b]^ For this purpose various strategies have been developed, which rely on the incorporation of non‐canonical amino acids that allow for subsequent chemical ligation like click chemistry. Incorporation of the required non‐canonical amino acids has mainly been achieved by selective pressure incorporation using auxotrophic strains,[^2b^, ^16^] or by stop codon suppression.[^2b^, ^17^] In comparison, the use of purified post‐translational modification (PTM) enzymes in combination with synthetic peptides – an approach also referred to as *in vitro* mutasynthesis^[18]^ – is a particularly elegant and versatile alternative to these strategies due to the possibility to introduce chemical variations at the level of the corepeptide without the need to adapt biological systems. In addition, the possibility to introduce functional tags prior to posttranslational tailoring, like in the case of biotinylation or dansylation has the huge advantage that postbiosynthetic chemical ligation becomes redundant. However, *in vitro* mutasynthesis approaches are often limited by the requirement of well‐characterized PTM enzymes with broad substrate tolerance that can be expressed and purified in an active soluble form.^[2b]^ Our results show, that two engineered ATP‐grasp ligases from the microviridin K biosynthesis pathway can be used *in vitro* to fully cyclize different non‐native core peptides decorated with a variety of non‐canonical amino acids with attractive properties like an affinity tag, a clickable handle or a fluorophore to tailor microviridin chemical probes, which alternatively would be hard to synthesize chemically from microviridin progenitors.

These results are of high relevance for RiPP engineering, since biosynthesis of the vast novel class of graspetides relies on homologues of the class‐defining macrolact(one/am)‐installing ATP‐grasp ligases used in this study. The graspetides gained increased attention recently after the discovery that macrocycle formation by ATP‐grasp ligases is widespread in RiPP biosynthesis with microviridins just being the tip of the iceberg of one of the fastest growing classes of RiPPs.[^2b^, ^6^, ^7^, ^19^] The discovery that plesiocin, thuringinin, and thatisin precursors also rely on dedicated motifs that are essential for recognition by the cognate ATP‐grasp ligases suggest that the chemoenzymatic strategy using modified core peptides together with leader peptide‐fused ATP‐grasp ligases could be expanded to the synthesis of other groups of graspetides.[^2b^, ^6a^, ^6b^]

Next, to check for bioactivity of the chemical probes, all tricyclic variants (**1**–**6**) were subjected to serine protease inhibition assays to determine IC_50_ values for microviridin J derivatives against trypsin, and for microviridin B derivatives against elastase. All modified compounds still show protease inhibition in the range of the unmodified control (IC_50_ values within one order of magnitude) except for dansylated microviridin B, which only retained partial activity (IC_50_ values bigger than one order of magnitude) (Table 1; Figures S38–S39).

**Table 1:** IC_50_ values for microviridin J and microviridin B derivatives.

Trypsin inhibition assay	IC_50_ [μM]	Elastase inhibition assay	IC_50_ [μM]
MvJ_CP_cy3_ (**1**)	1.09±0.09	MvB_CP_cy3_ (**4**)	0.33±0.02
MvJ_CP_N(Bio)_cy3_ (**2**)	3.53±0.30	MvB_CP_N(Bio)_cy3_ (**5**)	1.24±0.64
MvJ_CP_N(Prop)_cy3_ (**3**)	1.64±0.12	MvB_CP_N(Dansyl)_cy3_ (**6**)	28.05±7.17

Naturally, microviridins act as competitive inhibitors that bind tightly to their corresponding proteases in a substrate‐like manner, for which turnover is excluded. For the interaction of trypsin and microviridin J for example a K_D_ value of 0.68 μM was determined.^[8]^ Therefore, the chemical probes that show protease inhibition in the range of the unmodified microviridin derivatives could be expected to work as molecular tools. However, since the activity of dansylated microviridin B is clearly compromised (Table 1), the compound's affinity for elastase might not be strong enough for diagnostic applications.

**Figure 4:**
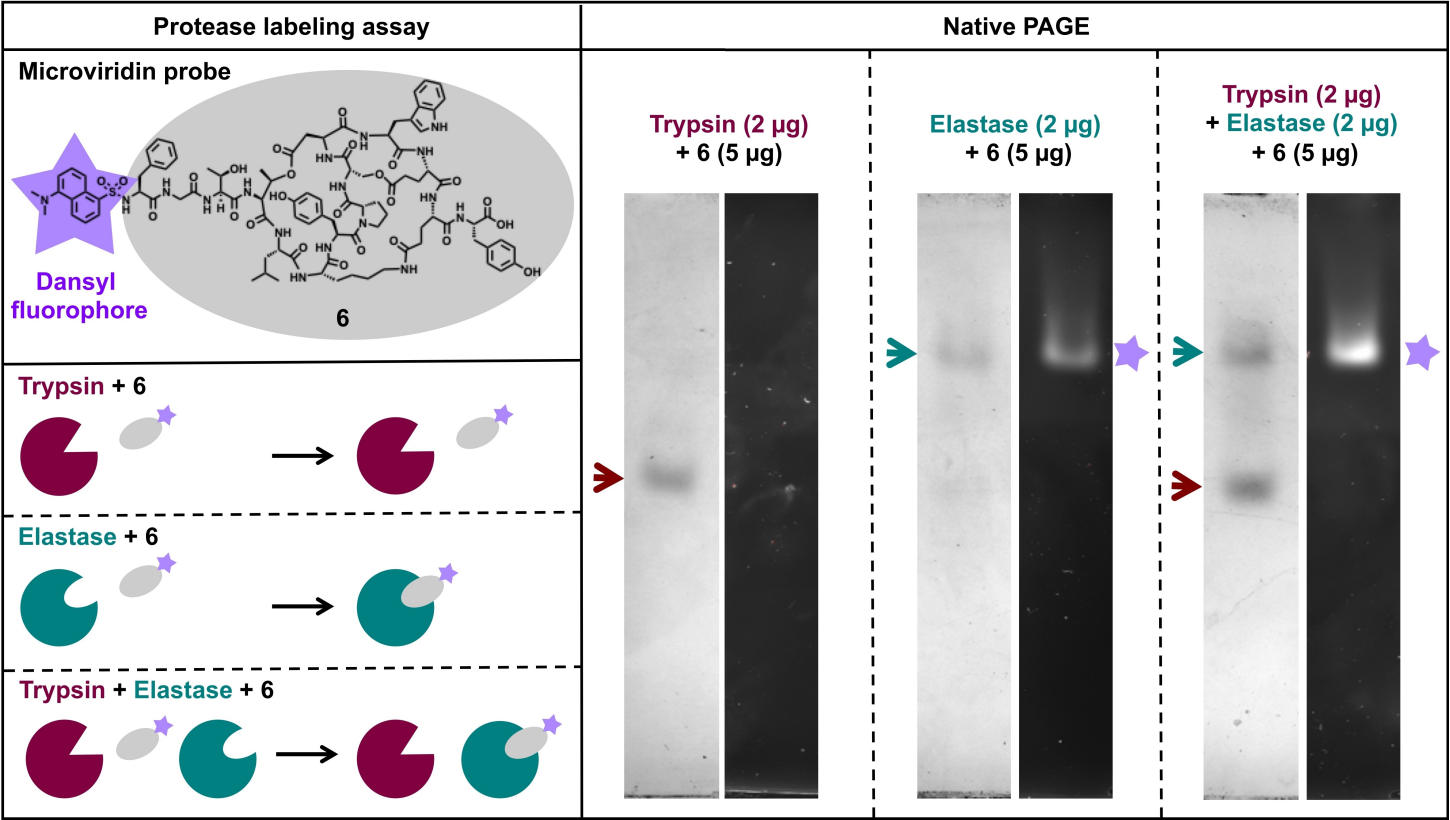
Protease labeling assay. The dansylated microviridin B probe (**6**) can be used as a molecular tool to selectively label small quantities of elastase in a protease mixture. This can be visualized by the fluorescence of the dansyl moiety, which exactly matches the protein bands corresponding to elastase in the native polyacrylamide gel. For a blank control see Figure S40.

To test whether the dansylated microviridin B probe can still be used as a molecular tool a native PAGE assay with trypsin and elastase was used. Native PAGE of elastase, trypsin and a mix of both preincubated with dansylated microviridin B clearly demonstrates that dansylated microviridin B can be used as a diagnostic tool *in vitro* to selectively label small quantities of elastase in a protease mixture (Figure 4). This experiment provides a proof of concept for the utility of microviridin chemical probes and highlights their potential even for derivatives whose activity might be compromised by introduced features.

## Conclusion

This study presents an elegant and versatile chemoenzymatic approach to introduce functional tags into microviridin natural products. Transformation of synthetic core peptides by constitutively activated ATP‐grasp ligases led to the synthesis of a series of N‐terminally modified microviridin J and microviridin B derivatives, which alternatively would be hard to synthesize chemically from microviridin progenitors. Remarkably, the biotinylated, dansylated or propargylated congeners retained full or at least partial activity against trypsin and elastase, respectively. Our work thus provides a small but targeted library of labeled protease inhibitors that can be used to explore the still elusive ecological functions and targets of this remarkable class of compounds and to advance applications based on protease inhibition, such as functionalized surfaces or drug conjugates.

## Experimental Section

**Synthesis of microviridin core peptides**: The peptide synthesis was carried out fully automated using a Prelude parallel synthesizer from Protein Technologies in 50 μmol scale. Coupling of Fmoc/*t*Bu‐protected amino acids was started using a tritylchloride polystyrene (TCP) resin and TBTU/DIPEA in DMF was used for coupling steps. All amino acid building blocks, including Fmoc‐l‐Tyr(Propargyl)‐OH and Fmoc‐l‐Lys(Biotin)‐OH, were purchased from Iris Biotech GmbH. Purification was performed on a preparative HPLC using an Agilent Technologies 1260 Series HPLC system equipped with an UV/Vis detector operating at λ=210 nm using a reverse phase Agilent C18 column (212×250 mm, particle size 10 μm). Characterization of the desired peptides was done by HR‐ESI‐MS on a Thermo Fisher Scientific LTQ Orbitrap XL. For a detailed synthesis protocol for automated solid‐phase peptide synthesis see the Supporting Information.

***In vitro*** **production of microviridins**: Enzyme production and purification, as well as microviridin cyclisation assays and product purification was carried out as previously reported,^[3]^ with the exception that the low background strain LOBSTR was used as *Escherichia coli* expression strain.^[20]^

**Mass spectrometric analysis of microviridins**: To confirm the modification state and cyclization of MvB and MvJ derivatives, reaction mixtures were separated by HPLC and relevant fractions, containing modified precursor peptides, were analyzed by mass spectrometry. Therefore, all HPLC fractions were first dried and subsequently redissolved in 20 μL of 50 % methanol. All MS and MS/MS measurement were conducted on a TimsTOF Flex MS‐system (Bruker Daltonics, Bremen, Germany) using the MALDI‐Ion source without utilization of the ion‐mobility unit. Samples were applied in the dried droplet method, as previously reported.^[4]^ Briefly 1 μL of each sample was mixed with an equal volume of the matrix solution (20 mg/mL 2,5‐DHB in 30 % acetonitrile, containing 0.1 % TFA) and subsequently 0.8 μL spotted on a MTP 384 ground steel target plate (Bruker). Measurements were conducted by acquiring 10,000 shots at a frequency of 10,000 Hz at a laser power of 80 %. Spectra were recorded in a mass range of 400–2,200 m/z after the system was calibrated using red phosphorous as a calibrant. Fragment spectra were recorded using collision induced dissociation (CID) with a fragmentation energy of 90 eV and an isolation width of 1.5 m/z. All spectra were subsequently analyzed using DataAnalysis 5.3 (Bruker).

**Protease inhibition assay**: Microviridins were checked for their inhibitory activity against trypsin and elastase. Therefore, microviridin concentrations of 0.01 μM, 0.1 μM, 1 μM, 10 μM and 100 μM were tested. A sample without microviridin was used as control. As substrates *N*_α_‐Benzoyl‐dl‐arginine 4‐nitroanilide hydrochloride (BAPNA) was used for the trypsin assay and *N*‐Succinyl‐Ala‐Ala‐Ala‐*p*‐nitroanilide (Suc‐AAA‐pNA) for the elastase assays. The trypsin assay was performed with 16.7 μg/mL (approximately 42.7 U/mL) bovine trypsin (Carl Roth (Germany)) and 108.5 μg/mL BAPNA (Sigma‐Aldrich (Germany)) in 50 mM Tris‐HCl, 20 mM CaCl_2_, 0.5 % DMSO, pH 7.5. The elastase assay was performed with 50 μg/mL (250 mU/mL) porcine pancreas elastase (Sigma‐Aldrich (Germany) and 500 μg/mL Suc‐AAA‐pNA (Sigma‐Aldrich (Germany)) in 50 mM Tris‐HCl, 20 mM CaCl_2_, pH 7.5. All microviridins were dissolved in water to a concentration of 500 μM except of tricyclic MvB_CP_N(dansyl), which was dissolved in water containing 10 % DMSO. Before adding the substrate, each enzyme was incubated for 5 min with the respective inhibitor at room temperature. Afterwards, the substrate was added, and the assays were incubated for 15 min (elastase assay) and 30 min (trypsin assay) at 37 °C. The enzymatic activity was measured spectrophotometrically (Varioscan Flash, Thermo Scientific) at 410 nm. The assays were done twice for each sample. IC50 values were calculated using the Quest Graph™ IC50 Calculator.^[21]^

**Protease labeling assay**: To test whether the dansylated microvoridin B variant (tricyclic MvB_CP_N(dansyl)) can selectively label elastase in a protease mixture 5 μg sample was mixed with 2 μg of elastase, 2 μg of trypsin and a mixture of both enzymes, respectively. Next, native loading dye was added (final concentration: 50 mM Tris, pH 6.8, 0.02 % bromophenol blue, 10 % glycerol) and samples were separated by native gel electrophoresis using Precast Mini‐PROTEAN TGX 8–16 % gels (Bio‐Rad). As running buffer, 10x Tris/Glycine Buffer (Bio‐Rad) was diluted to a 1x concentration. The gel was run with 150 V for 3 hours at 4 °C. Fluorescence images were taken with the ChemiDoc XRS+ Imaging System (Bio‐Rad) using the ethidium bromide settings. Afterwards, the gel was stained with Coomassie staining solution (5 % (w/v) aluminium sulfate, 10 % (v/v) ethanol, 0.02 % (w/v) CBB, 2 % (v/v) phosphoric acid) over night and subsequently destained with destaining solution (2 % (v/v) phosphoric acid, 10 % (v/v) ethanol). Images were taken with the ChemiDoc XRS+ Imaging System (Bio‐Rad) using the Coomassie settings.

## Conflict of interest

The authors declare no conflict of interest.

1

## Supporting information

As a service to our authors and readers, this journal provides supporting information supplied by the authors. Such materials are peer reviewed and may be re‐organized for online delivery, but are not copy‐edited or typeset. Technical support issues arising from supporting information (other than missing files) should be addressed to the authors.

Supporting Information

Supporting Information

## Data Availability

The data that support the findings of this study are available from the corresponding author upon reasonable request.
