# Supplementary material for: One‐Pot Chemoenzymatic Synthesis of Microviridin Analogs Containing Functional Tags
Source: Chembiochem. 2022 Sep 13;23(20):e202200345. doi: 10.1002/cbic.202200345 (PMC9826346; doi:10.1002/cbic.202200345)
Supplement: Supplementary file 1 — Supporting Information [file CBIC-23-0-s001.pdf]

ChemBioChem

Supporting Information

One-Pot Chemoenzymatic Synthesis of Microviridin Analogs Containing Functional Tags

Stella Scholz, Sofia Kerestetzopoulou, Vincent Wiebach, Romina Schnegotzki, Bianca Schmid, Emmanuel Reyna-González, Ling Ding, Roderich D. Süssmuth, Elke Dittmann, and Martin Baunach*

1. Material and Methods (Aditional Notes)

2. Supplementary Tables

Table S1. Mass spectrometric analysis of the cyclization assays.
Table S2. Overall theoretical yields, isolated yields and percent yields for the enzymatic conversions.

3. Supplementary Figures

Figure S1. Chemoenzymatic synthesis of C-terminally modified Mv J and Mv B derivatives.
Figure S2. Results of the the repeated enzymatic conversion of MvJ_CP_N(Prop).
Figure S3. MALDI-TOF MS spectrum of unmodified MvJ_CP.
Figure S4. MALDI-TOF MS spectrum of bicyclic MvJ_CP.
Figure S5. MALDI-TOF MS spectrum of tricyclic MvJ_CP.
Figure S6. MALDI-TOF MS spectrum of unmodified MvJ_CP_N(Bio).
Figure S7. MALDI-TOF MS spectrum of bicyclic MvJ_CP_N(Bio).
Figure S8. MALDI-TOF MS spectrum of tricyclic MvJ_CP_N(Bio).
Figure S9. MALDI-TOF MS spectrum of unmodified MvJ_CP_C(Bio).
Figure S10. MALDI-TOF MS spectrum of monocyclic MvJ_CP_C(Bio).
Figure S11. MALDI-TOF MS spectrum of unmodified MvJ_CP_N(Prop).
Figure S12. MALDI-TOF MS spectrum of monocyclic MvJ_CP_N(Prop).
Figure S13. MALDI-TOF MS spectrum of bicyclic MvJ_CP_N(Prop).
Figure S14. MALDI-TOF MS spectrum of tricyclic MvJ_CP_N(Prop).
Figure S15. MALDI-TOF MS spectrum of unmodified MvJ_CP_C(Prop).
Figure S16. MALDI-TOF MS spectrum of monocyclic MvJ_CP_C(Prop).
Figure S17. MALDI-TOF MS spectrum of unmodified MvB_CP.
Figure S18. MALDI-TOF MS spectrum of monocyclic MvB_CP.
Figure S19. MALDI-TOF MS spectrum of bicyclic MvB_CP.
Figure S20. MALDI-TOF MS spectrum of tricyclic MvB_CP.
Figure S21. MALDI-TOF MS spectrum of unmodified MvB_CP_N(Bio).
Figure S22. MALDI-TOF MS spectrum of monocyclic MvB_CP_N(Bio).
Figure S23. MALDI-TOF MS spectrum of bicyclic MvB_CP_N(Bio).
Figure S24. MALDI-TOF MS spectrum of tricyclic MvB_CP_N(Bio).
Figure S25. MALDI-TOF MS spectrum of unmodified MvB_CP_C(Bio).
Figure S26. MALDI-TOF MS spectrum of monocyclic MvB_CP_C(Bio).
Figure S27. MALDI-TOF MS spectrum of unmodified MvB_CP_N(dansyl).
Figure S28. MALDI-TOF MS spectrum of monocyclic MvB_CP_N(dansyl).
Figure S29. MALDI-TOF MS spectrum of bicyclic MvB_CP_N(dansyl).
Figure S30. MALDI-TOF MS spectrum of tricyclic MvB_CP_N(dansyl).
Figure S31. MALDI-TOF MS/MS spectrum of tricyclic MvJ_CP (**1**).
Figure S32. MALDI-TOF MS/MS spectrum of tricyclic MvJ_CP_N(Bio) (**2**).
Figure S33. MALDI-TOF MS/MS spectrum of tricyclic MvJ_CP_N(Prop) (**3**).
Figure S34. MALDI-TOF MS/MS spectrum of tricyclic MvB_CP (**4**).
Figure S35. MALDI-TOF MS/MS spectrum of tricyclic MvB_CP_N(Bio) (**5**).
Figure S36. MALDI-TOF MS/MS spectrum of tricyclic MvB_CP_N(dansyl) (**6**).
Figure S37. Ring-opening reactions during MS/MS analysis.
Figure S38. Results of the protease inhibition assay of the microviridin J derivatives.
Figure S39. Results of the protease inhibition assay of the microviridin B derivatives.
Figure S40. Blank control for protease labeling assay.

1. Material and Methods (Addional Notes)

Synthesis protocol for automated solid-phase peptide synthesis

Loading:
To a 20 ml syringe reactor with frit and cap 2-chloro-tritylchloride (TCP) resin (2 g, 1.4 mmol/g) and dry DCM (14 mL) were added. The resin was pre-swollen for 10 min and the solvent was removed by evaporation in vacuum. A mixture of the amino acid (8.40 mmol, 3 eq.) and DIPEA (5 eq.) dissolved in dry DCM (10 mL) was added to the resin. The syringe was agitated for 30 min at room temperature. The solution was removed and the resin was washed (2x 10 mL DMF, 1x 10 mL DCM). Capping of non-reacted functional groups of the resin was performed with DCM, methanol and DIPEA 80:15:5 (2x 15 mL, 10 min). After washing (5x 10 mL DMF), Fmoc-removal was achieved with DMF/piperidine (4:1, 10 mL, 1x 2 min, 1x 20 min). After final washing (2x 10 mL DMF, 1x 10 mL methanol, 3x 10 ml DCM), the resin was dried *in vacuo*.
Coupling of Fmoc/*t*Bu-protected amino acids:
To 200 mg of the resin (~ 0.5 mmol/g), a 0.25 M solution of the amino acid in DMF (2.5 eq. relative to resin loading) was added. After addition of a 0.5 M solution of DIPEA in DMF (2.5 eq.) and a 0.25 M solution of TBTU in DMF (2.5 eq.), the reaction solution was mixed for 15 min. A second coupling was performed for 15 min. For couplings subsequent to the 5th amino acid, double couplings with 30 min coupling time were performed. For couplings subsequent to the 10th amino acid, a third coupling with 45 min was performed. After each coupling cycle capping with 0.5 M acetic anhydride in DMF (2 x 2.5 mL, 10 min) was performed. Finally, the resin was washed with DMF (6 x 2.5 mL).
Fmoc removal:
DMF/piperidine (4:1, 2.5 mL) was added to the resin and mixed for 2.5 min. The procedure was repeated 4 times. The resin was washed with DMF (6x 2.5 mL). After the final coupling cycle, the resin was washed with DCM (3 x 2 ml).
Dansyl derivatization:
The reagent 1-dimethylaminonaphthalene-5-sulfonyl chloride (dansyl chloride, DNS-Cl, 3eq.) and DIPEA (3 eq.) in DMF (10 mL) were added to the resin-bound peptide after Fmoc-removal to derivatize the N-terminal free amino group. The suspension was mixed for 20 min. The resin was washed with DMF (3x 2.5 mL).
Global deprotection:
The resin was transferred to a 5 mL syringe with frit and cap. After addition of the cleavage cocktail (TFA, H_2O, TES, DODT (3,6-dioxa-1,8-octane-dithiole) 92.5:2.5:2.5:2.5), the syringe was shaken for 3 h. The peptide was precipitated in ice cold diethyl ether and centrifuged. The supernatant was removed and the precipitate was washed with diethyl ether twice. The peptide was resolved in MeCN/H_2O (1:4) and lyophilized.

2. Supplementary Tables

Table S1. Mass spectrometric analysis of the cyclization assays. The masses of the modified core peptides correspond to the monocyclic, bicyclic and tricyclic microviridin products due to mass shifts suggestive of the loss of one, two, or three water molecules ($-H_2O$: mass shift of 18.01 Da) as a consequence of lactone and lactame ring forming during condensation reactions.

		Calculated (Da)	Measured (m/z)	Error [ppm]
**MvJ_CP**	Unmodified	1696.7966	1696.8045	4.7
	Bicyclic	1660.7755	1660.7842	5.3
	Tricyclic	1642.7649	1642.7729	4.9
**MvJ_CP_N(Bio)**	Unmodified	2050.9692	2050.9781	4.4
	Bicyclic	2014.9480	2014.9561	4.0
	Tricyclic	1996.9375	1996.9462	4.4
**MvJ_CP_C(Bio)**	Unmodified	2050.9692	2050.9778	4.2
	Monocyclic	2032.9586	2032.9656	3.4
**MvJ_CP_N(Prop)**	Unmodified	1897.8756	1897.8817	3.2
	Monocyclic	1879.8671	1879.8702	2.8
	Bicyclic	1861.8545	1861.8629	4.5
	Tricyclic	1843.8439	1843.8489	2.7
**MvJ_CP_C(Prop)**	Unmodified	1897.8756	1897.8827	4.2
	Monocyclic	1879.8650	1897.8827	3.4
**MvB_CP**	Unmodified	1735.7850	1735.7894	2.5
	Monocyclic	1717.7745	1717.7776	1.8
	Bicyclic	1699.7639	1699.7692	3.1
	Tricyclic	1681.7534	1681.7576	2.5
**MvB_CP_N(Bio)**	Unmodified	2089.9576	2089.9606	1.4
	Monocyclic	2071.9470	2071.9515	2.2
	Bicyclic	2053.9365	2053.9425	2.9
	Tricyclic	2035.9259	2035.9342	4.1
**MvB_CP_C(bio)**	Unmodified	2089.9576	2089.9661	4.1
	Monocyclic	2071.9470	2071.9563	4.5
**MvB_CP_N(dansyl)**	Unmodified	1968.8361	1968.8436	3.8
	Monocyclic	1950.8255	1950.8321	3.4
	Bicyclic	1932.8150	1932.8233	4.3
	Tricyclic	1914.8044	1914.8120	4.0

Table S2. Overall theoretical yields, isolated yields and percent yields for the enzymatic conversions.

	**Theoretical yield**	**Isolated yield**	**Percent yield**
MvJ_CP$_{cy3}$ (**1**)	1549 µg	302 µg	19%
MvJ_CP_N(Bio)$_{cy3}$ (**2**)	1558 µg	213 µg	14%
MvJ_CP_N(Prop)$_{cy3}$ (**3**)	2798 µg	158 µg	6%
MvB_CP$_{cy3}$ (**4**)	1440 µg	272 µg	19%
MvB_CP_N(Bio)$_{cy3}$ (**5**)	1559 µg	243 µg	16%
MvB_CP_N(dansyl)$_{cy3}$ (**6**)	1556 µg	398 µg	26%

3. Supplementary Figures

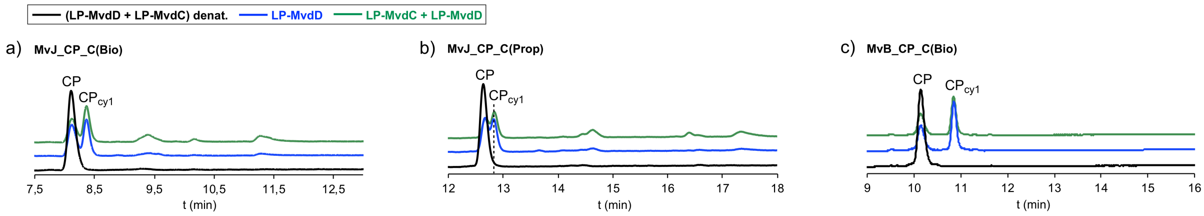

Figure S1. Chemoenzymatic synthesis of C-terminally modified microviridin J and microviridin B derivatives by using LP-MvdD alone (blue line) or in combination with LP-MvdC (green line). a)-c) HPLC monitoring (absorbance at 199 nm) of enzyme assays. Novel peaks correspond to monocyclic (CP_{cy1}) microviridin products as indicated by mass spectrometric analysis (Figure S3-S30, Table S1).

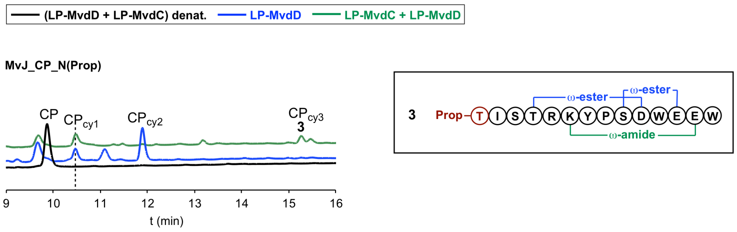

Figure S2. Results of the the repeated enzymatic conversion of MvJ_CP_N(Prop). HPLC monitoring (absorbance at 199 nm) of enzyme assay.

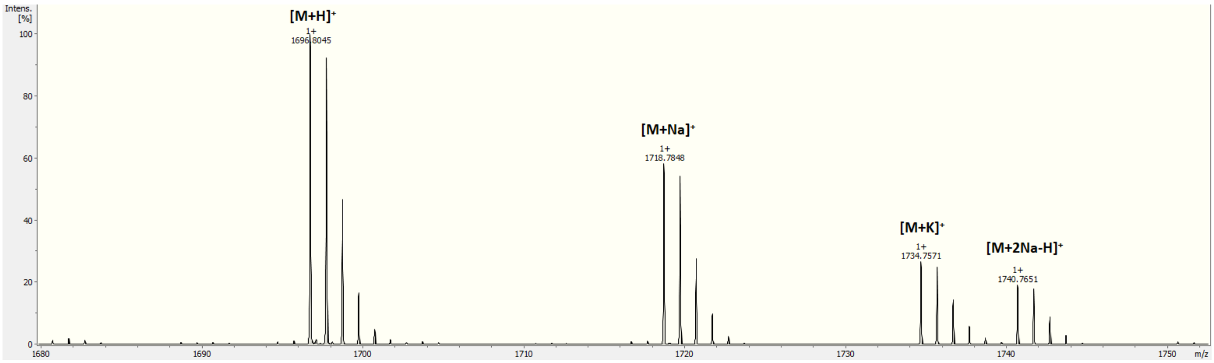

Figure S3. MALDI-TOF MS spectrum of unmodified MvJ_CP with relevant adduct ions labelled. Overview of measured and calculated masses can be found in Table S1.

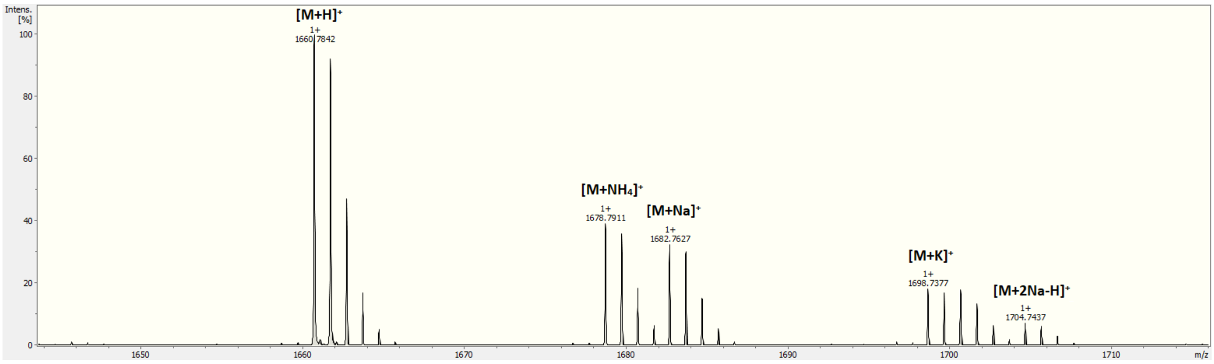

Figure S4. MALDI-TOF MS spectrum of bicyclic MvJ_CP with relevant adduct ions labelled. Overview of measured and calculated masses can be found in Table S1.

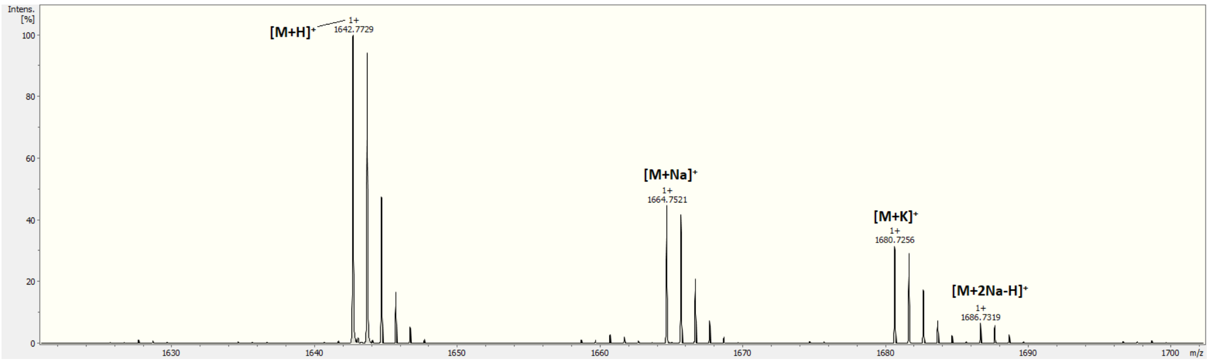

Figure S5. MALDI-TOF MS spectrum of tricyclic MvJ_CP with relevant adduct ions labelled. Overview of measured and calculated masses can be found in Table S1.

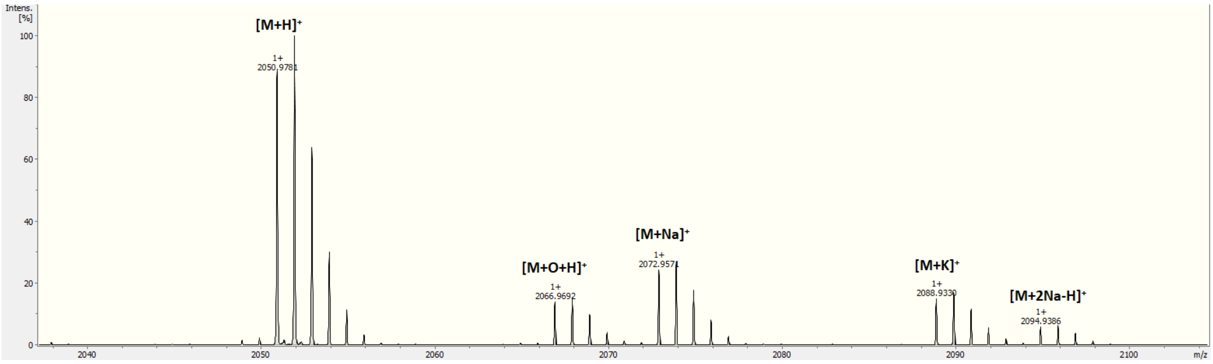

Figure S6. MALDI-TOF MS spectrum of unmodified MvJ_CP_N(Bio) with relevant adduct ions labelled. Overview of measured and calculated masses can be found in Table S1.

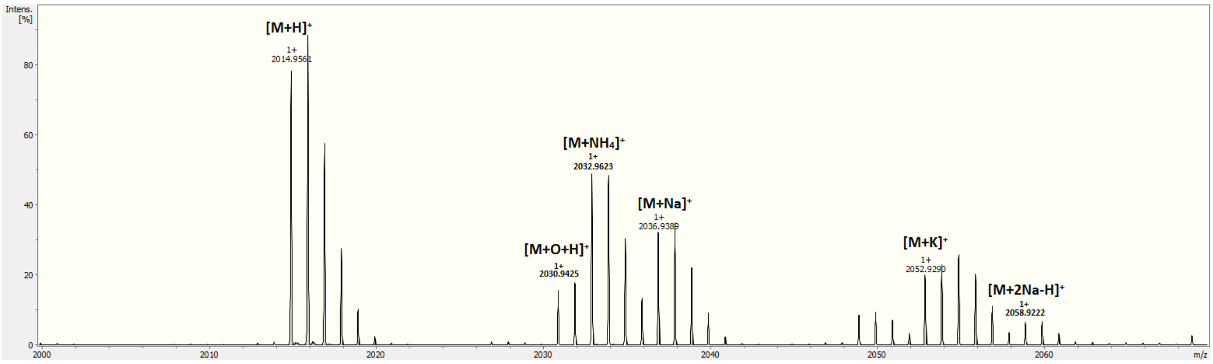

Figure S7. MALDI-TOF MS spectrum of bicyclic MvJ_CP_N(Bio) with relevant adduct ions labelled. Overview of measured and calculated masses can be found in Table S1.

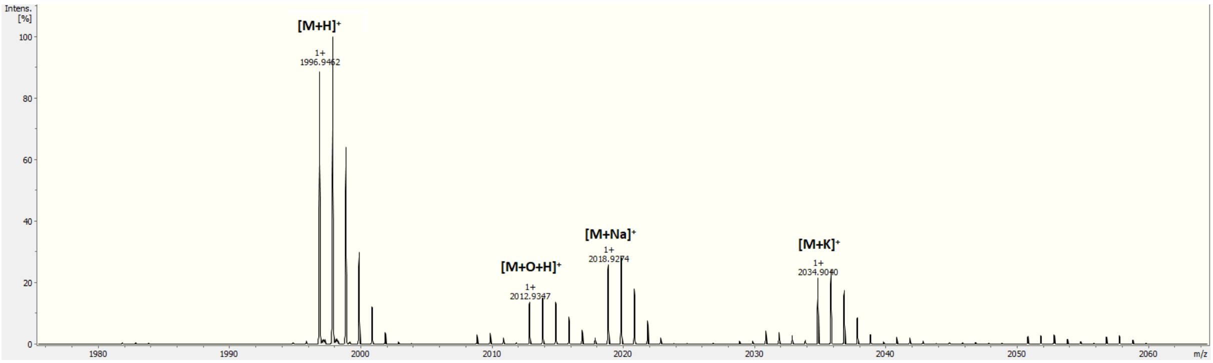

Figure S8. MALDI-TOF MS spectrum of tricyclic MvJ_CP_N(Bio) with relevant adduct ions labelled. Overview of measured and calculated masses can be found in Table S1.

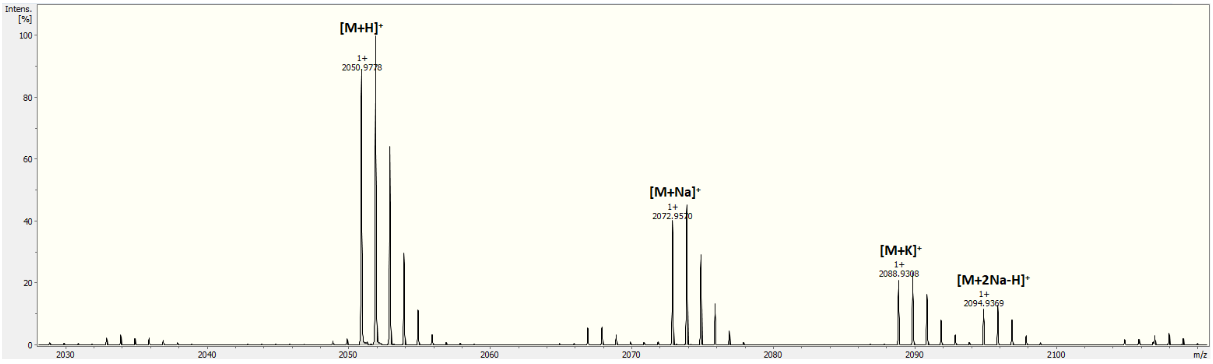

Figure S9. MALDI-TOF MS spectrum of unmodified MvJ_CP_C(Bio) with relevant adduct ions labelled. Overview of measured and calculated masses can be found in Table S1.

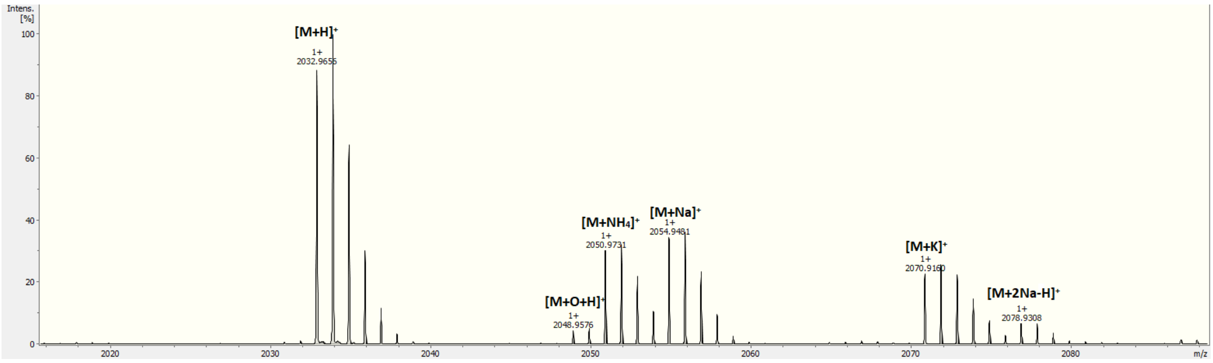

Figure S10. MALDI-TOF MS spectrum of monocyclic MvJ_CP_C(Bio) with relevant adduct ions labelled. Overview of measured and calculated masses can be found in Table S1.

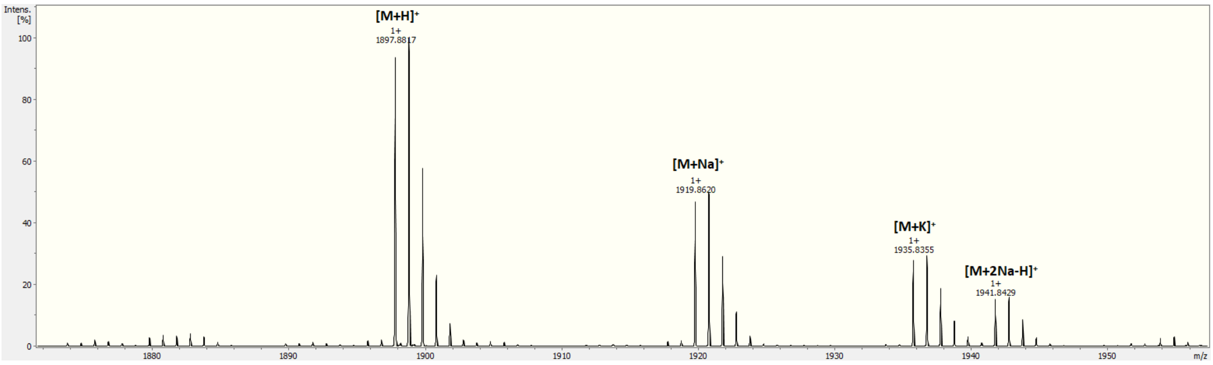

Figure S11. MALDI-TOF MS spectrum of unmodified MvJ_CP_N(Prop) with relevant adduct ions labelled. Overview of measured and calculated masses can be found in Table S1.

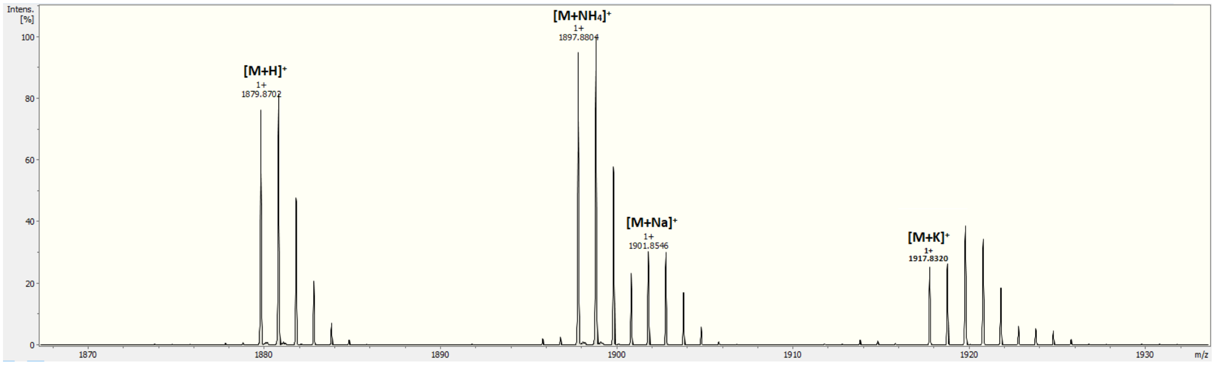

Figure S12. MALDI-TOF MS spectrum of monocyclic MvJ_CP_N(Prop) with relevant adduct ions labelled. Overview of measured and calculated masses can be found in Table S1.

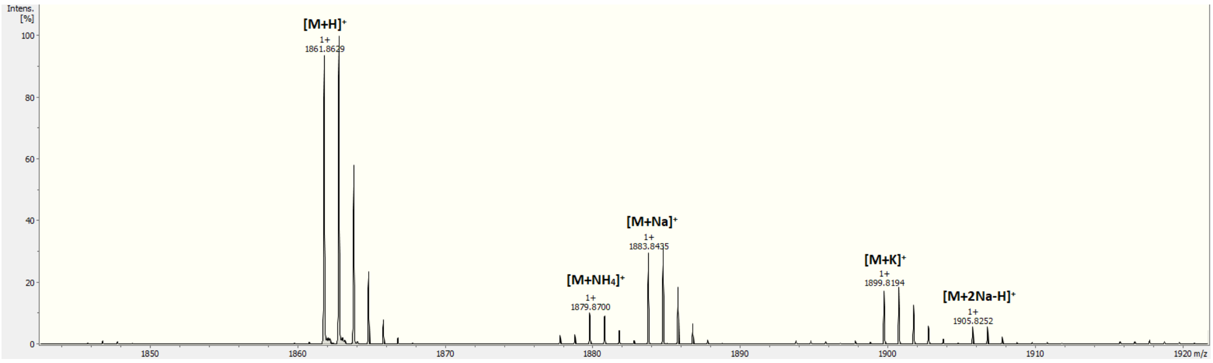

Figure S13. MALDI-TOF MS spectrum of bicyclic MvJ_CP_N(Prop) with relevant adduct ions labelled. Overview of measured and calculated masses can be found in Table S1.

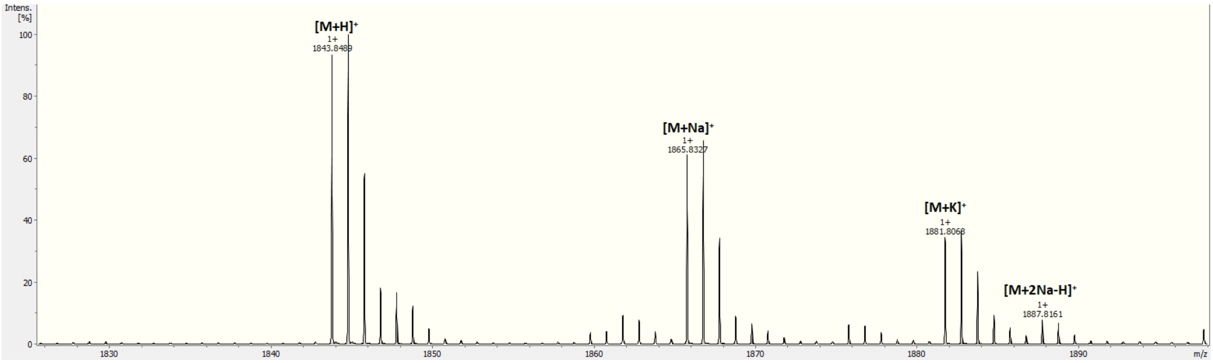

Figure S14. MALDI-TOF MS spectrum of tricyclic MvJ_CP_N(Prop) with relevant adduct ions labelled. Overview of measured and calculated masses can be found in Table S1.

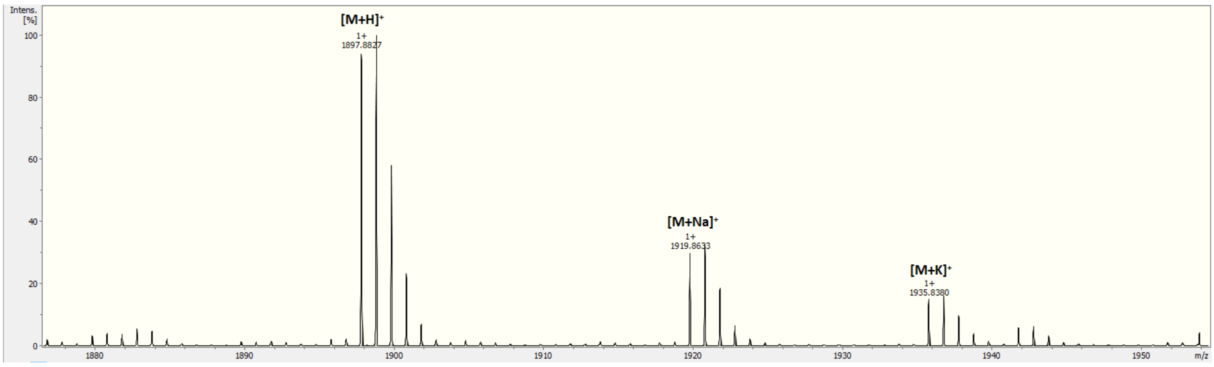

Figure S15. MALDI-TOF MS spectrum of unmodified MvJ_CP_C(Prop) with relevant adduct ions labelled. Overview of measured and calculated masses can be found in Table S1.

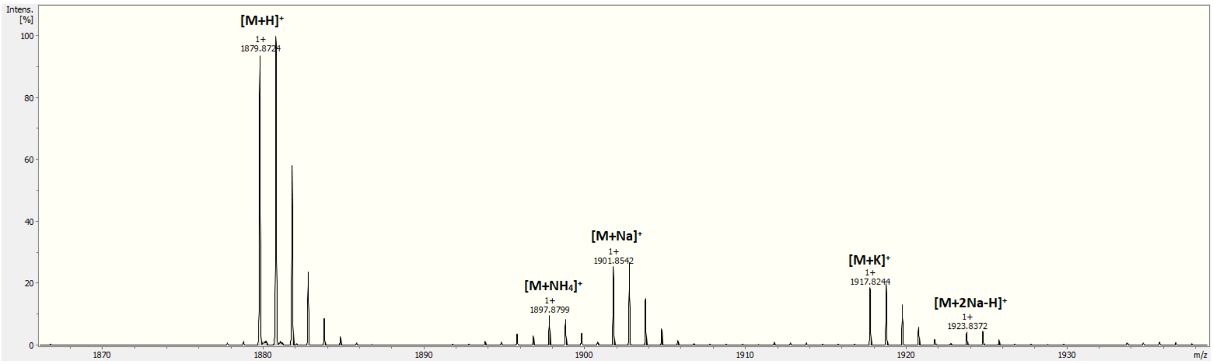

Figure S16. MALDI-TOF MS spectrum of monocyclic MvJ_CP_C(Prop) with relevant adduct ions labelled. Overview of measured and calculated masses can be found in Table S1.

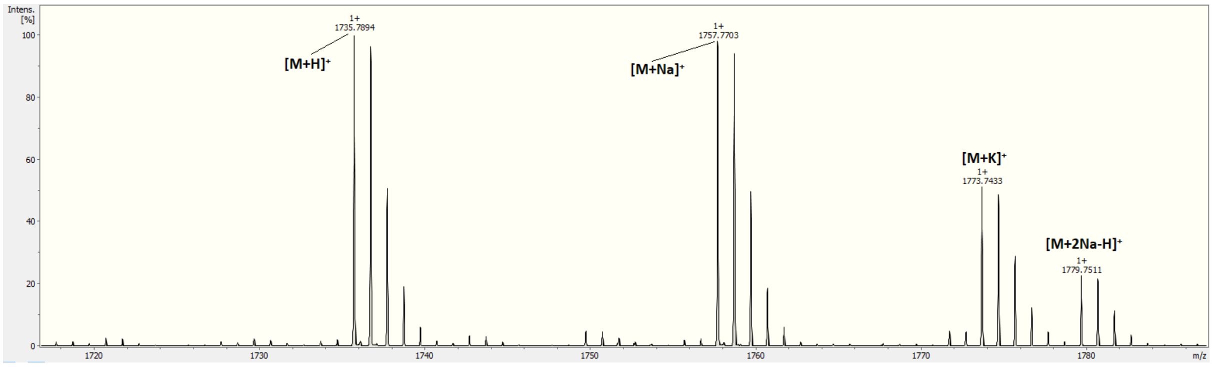

Figure S17. MALDI-TOF MS spectrum of unmodified MvB_CP with relevant adduct ions labelled. Overview of measured and calculated masses can be found in Table S1.

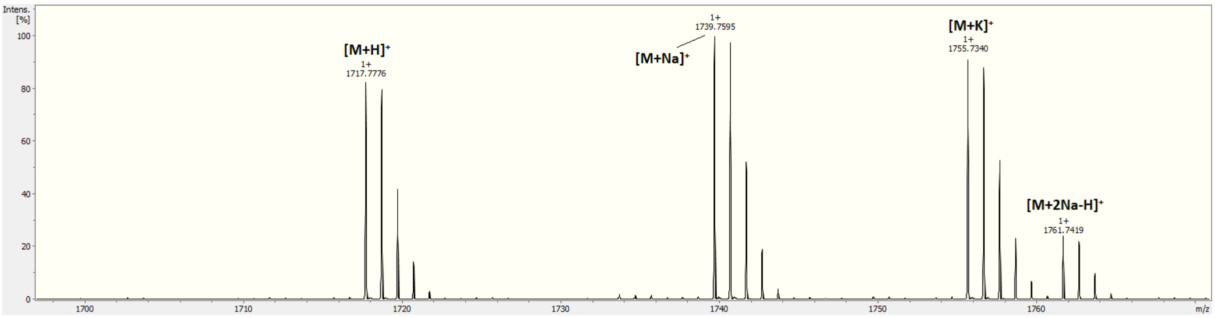

Figure S18. MALDI-TOF MS spectrum of monocyclic MvB_CP with relevant adduct ions labelled. Overview of measured and calculated masses can be found in Table S1.

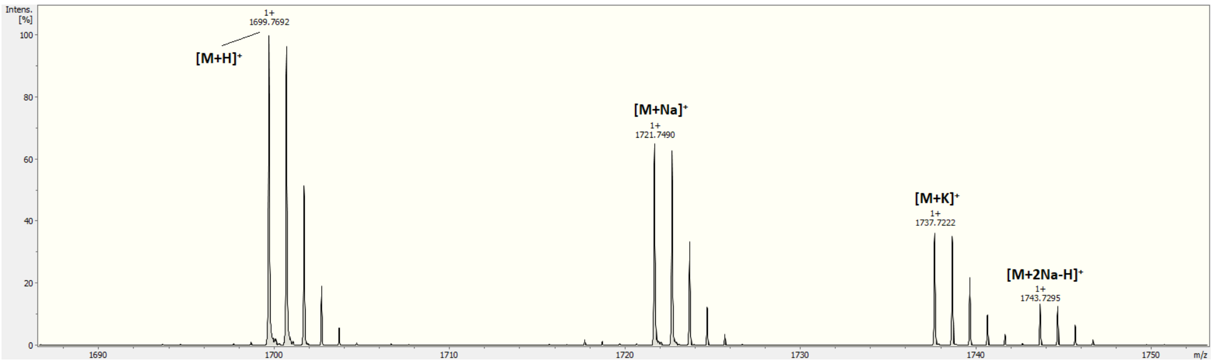

Figure S19. MALDI-TOF MS spectrum of bicyclic MvB_CP with relevant adduct ions labelled. Overview of measured and calculated masses can be found in Table S1.

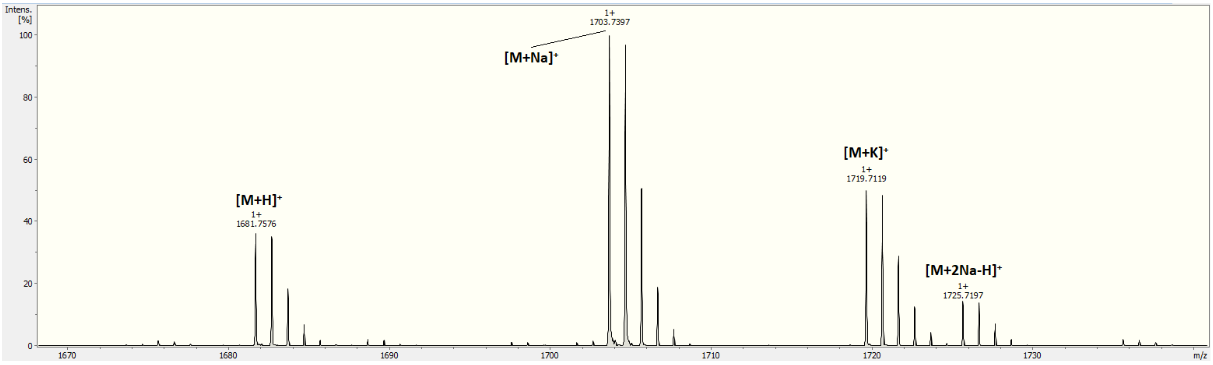

Figure S20. MALDI-TOF MS spectrum of tricyclic MvB_CP with relevant adduct ions labelled. Overview of measured and calculated masses can be found in Table S1.

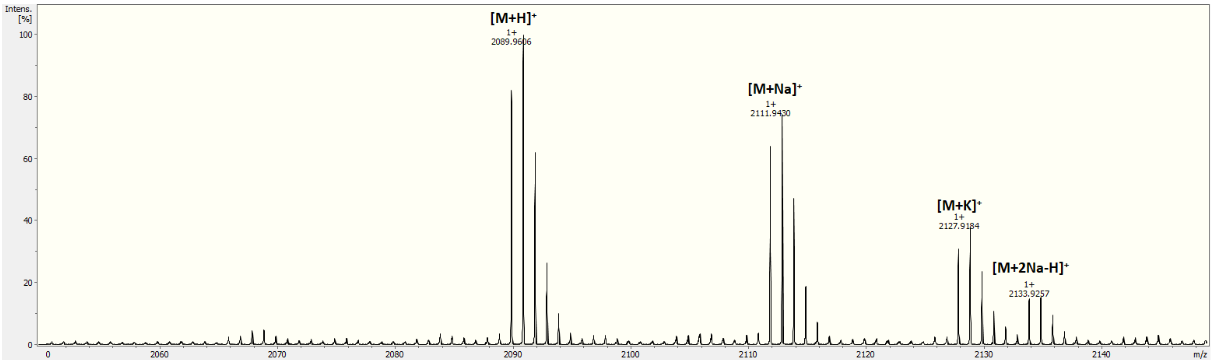

Figure S21. MALDI-TOF MS spectrum of unmodified MvB_CP_N(Bio) with relevant adduct ions labelled. Overview of measured and calculated masses can be found in Table S1.

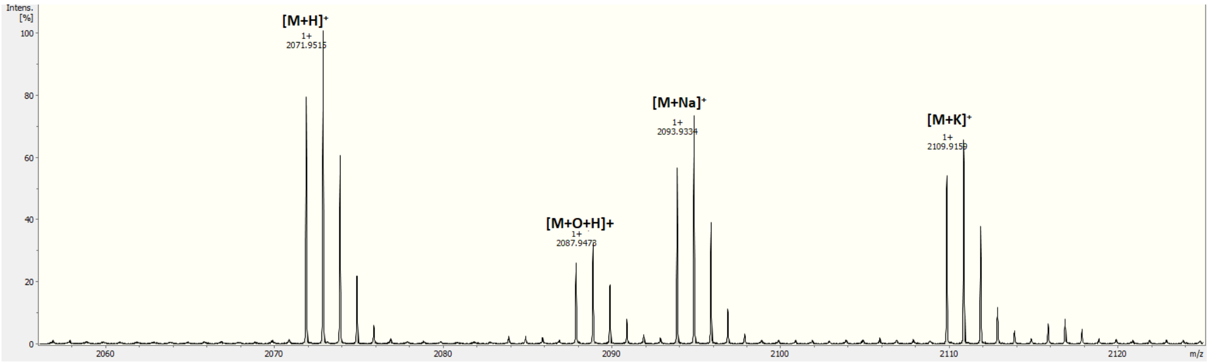

Figure S22. MALDI-TOF MS spectrum of monocyclic MvB_CP_N(Bio) with relevant adduct ions labelled. Overview of measured and calculated masses can be found in Table S1.

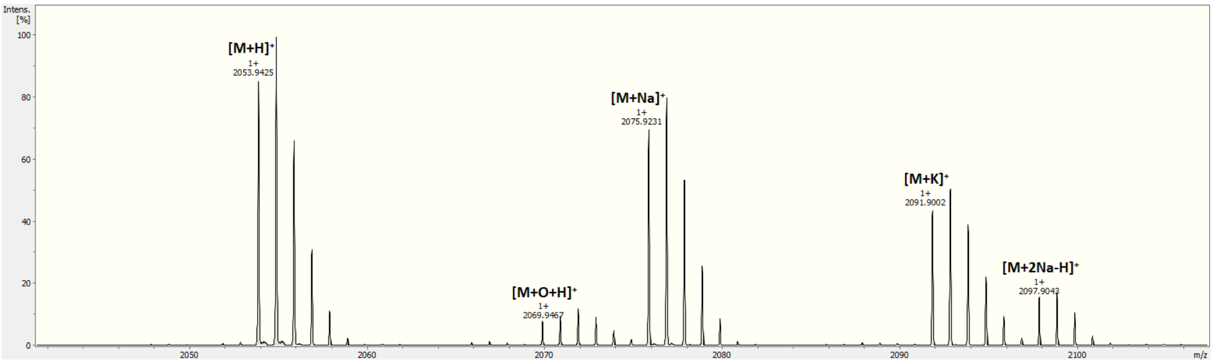

Figure S23. MALDI-TOF MS spectrum of bicyclic MvB_CP_N(Bio) with relevant adduct ions labelled. Overview of measured and calculated masses can be found in Table S1.

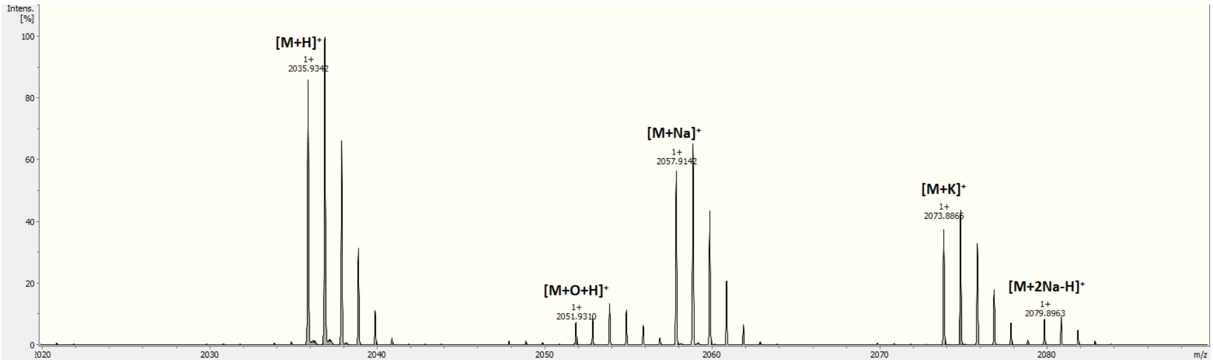

Figure S24. MALDI-TOF MS spectrum of tricyclic MvB_CP_N(Bio) with relevant adduct ions labelled. Overview of measured and calculated masses can be found in Table S1.

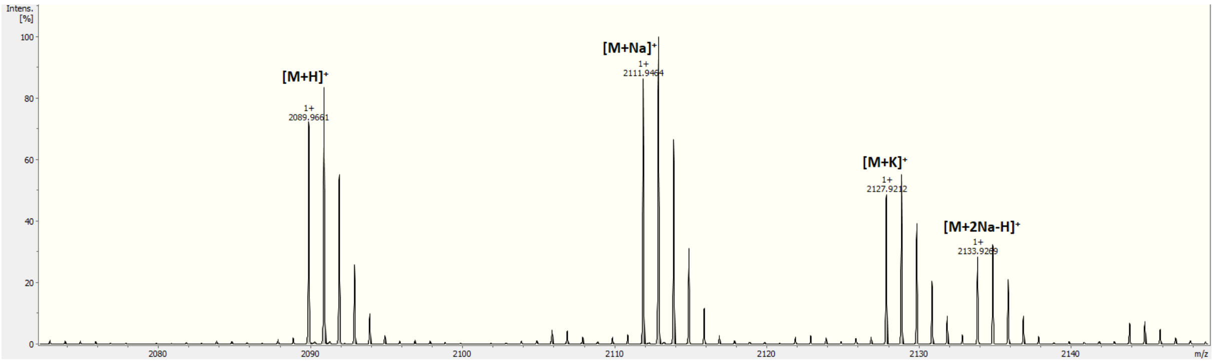

Figure S25. MALDI-TOF MS spectrum of unmodified MvB_CP_C(Bio) with relevant adduct ions labelled. Overview of measured and calculated masses can be found in Table S1.

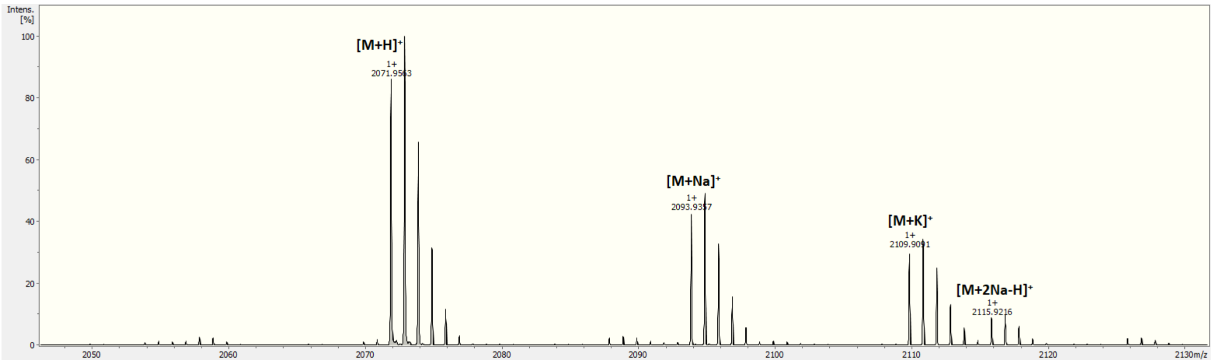

Figure S26. MALDI-TOF MS spectrum of monocyclic MvB_CP_C(Bio) with relevant adduct ions labelled. Overview of measured and calculated masses can be found in Table S1.

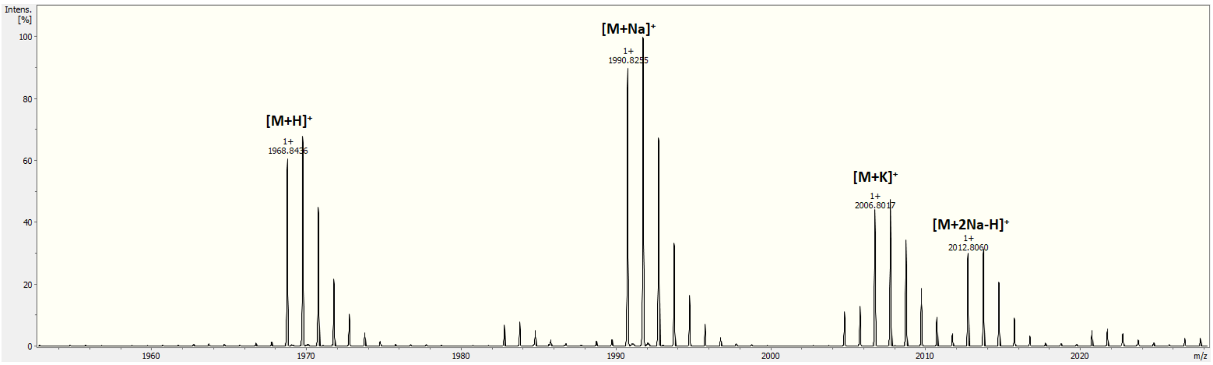

Figure S27. MALDI-TOF MS spectrum of unmodified MvJ_CP_N(dansyl) with relevant adduct ions labelled. Overview of measured and calculated masses can be found in Table S1.

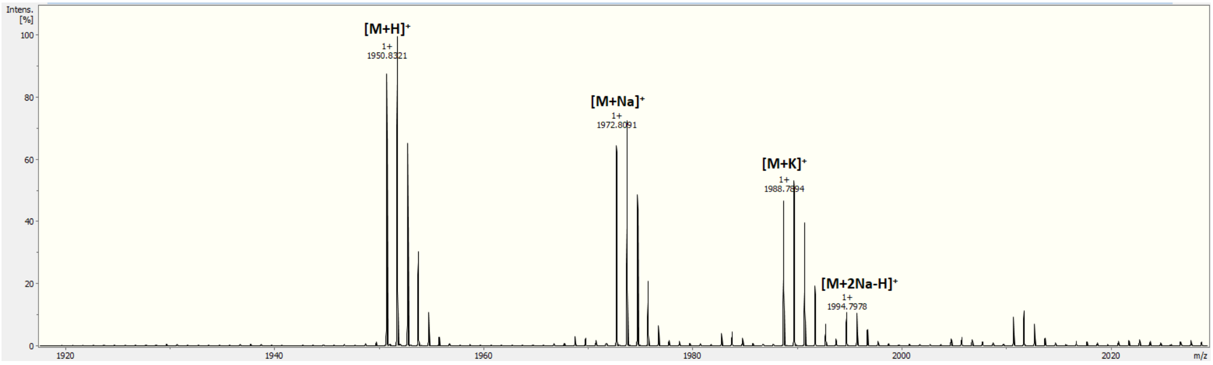

Figure S28. MALDI-TOF MS spectrum of monocyclic MvJ_CP_N(dansyl) with relevant adduct ions labelled. Overview of measured and calculated masses can be found in Table S1.

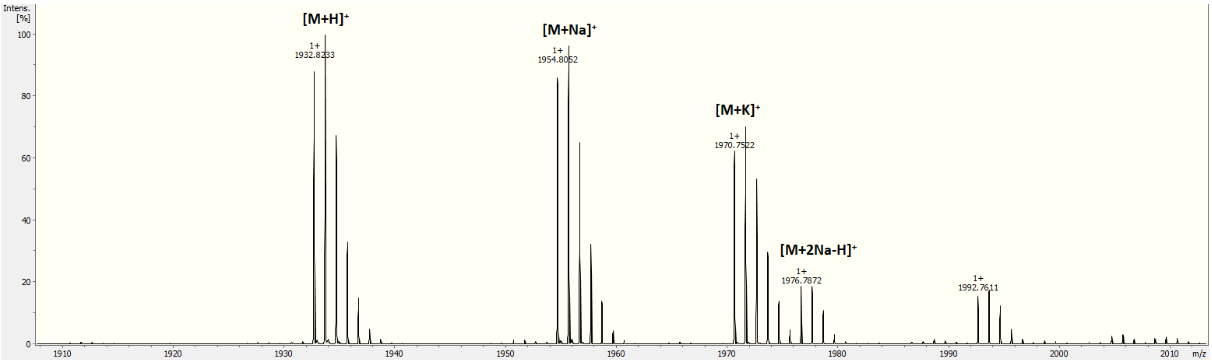

Figure S29. MALDI-TOF MS spectrum of bicyclic MvJ_CP_N(dansyl) with relevant adduct ions labelled. Overview of measured and calculated masses can be found in Table S1.

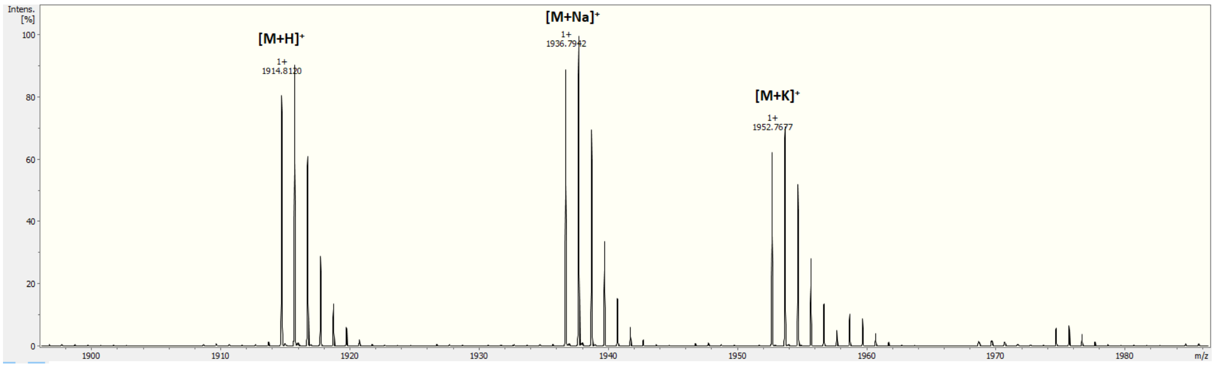

Figure S30. MALDI-TOF MS spectrum of tricyclic MvJ_CP_N(dansyl) with relevant adduct ions labelled. Overview of measured and calculated masses can be found in Table S1.

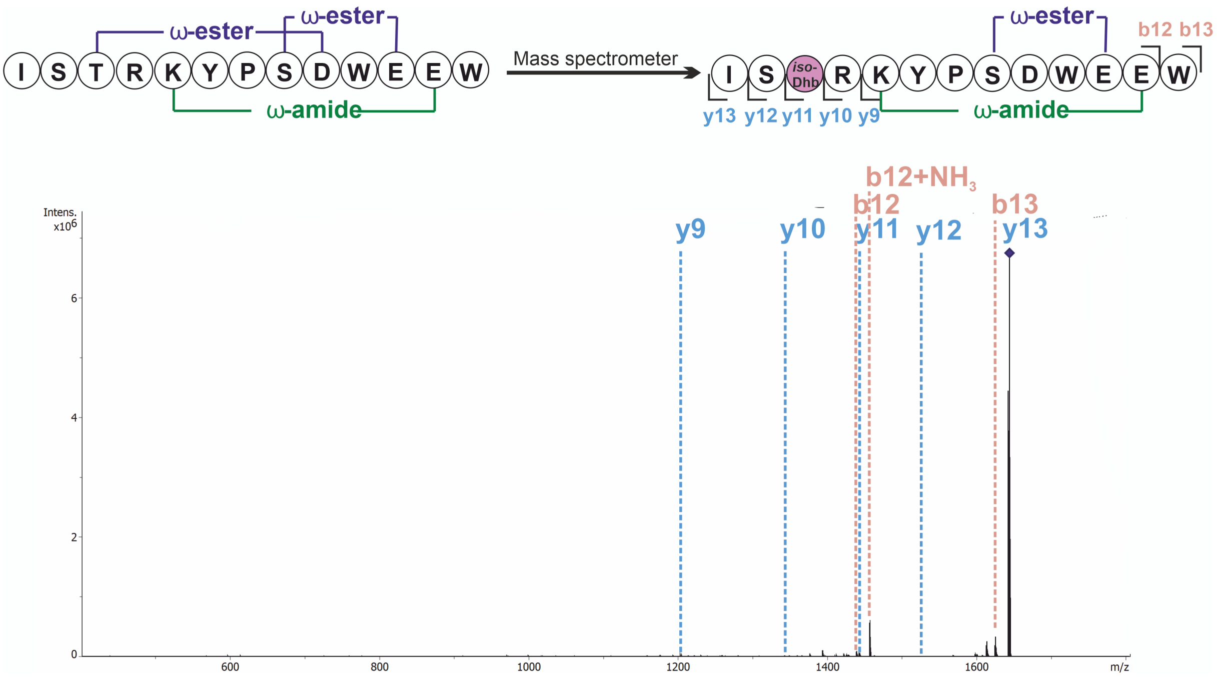

Figure S31. MALDI-TOF MS/MS spectrum of tricyclic MvJ_CP (microviridin J core peptide) (**1**), which shows a fragmentation pattern diagnostic for the tricyclic architecture of microviridins. As previously reported,[1] the N-terminal lactone ring of microviridin opens during MALDI-TOF MS/MS analysis due to a rearrangement reaction, yielding a dehydrated threonine (*iso*-dehydrobutyrine; *iso*-Dhb) and the carboxylic acid moiety of aspartate instead of a lactone bond (Fig. S37). For a comprehensive list of all calculated and observed fragment ions see the supplementary Excel file.

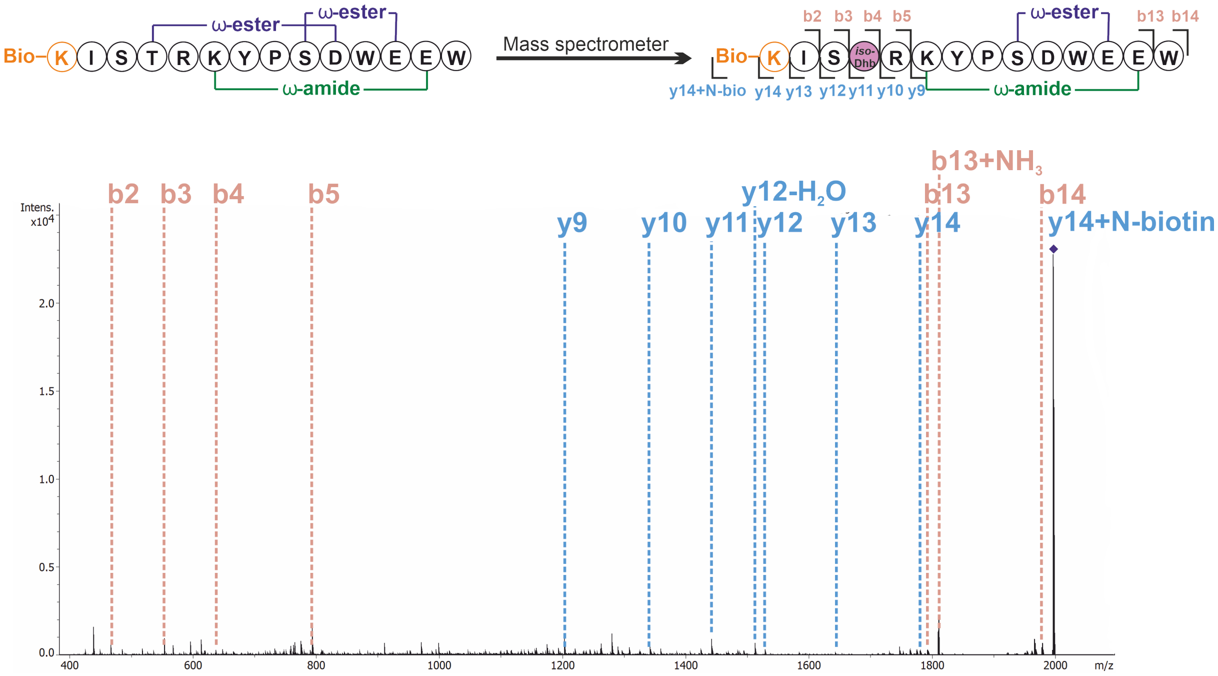

Figure S32. MALDI-TOF MS/MS spectrum of tricyclic MvJ_CP_N(Bio) (microviridin J core peptide with N-terminal N^{ε}-biotinyl-L-lysine) (**2**), which shows a fragmentation pattern diagnostic for the tricyclic architecture of microviridins. As previously reported,[1] the N-terminal lactone ring of microviridin opens during MALDI-TOF MS/MS analysis due to a rearrangement reaction, yielding a dehydrated threonine (*iso*-dehydrobutyrine; *iso*-Dhb) and the carboxylic acid moiety of aspartate instead of a lactone bond (Fig. S37). For a comprehensive list of all calculated and observed fragment ions see the supplementary Excel file.

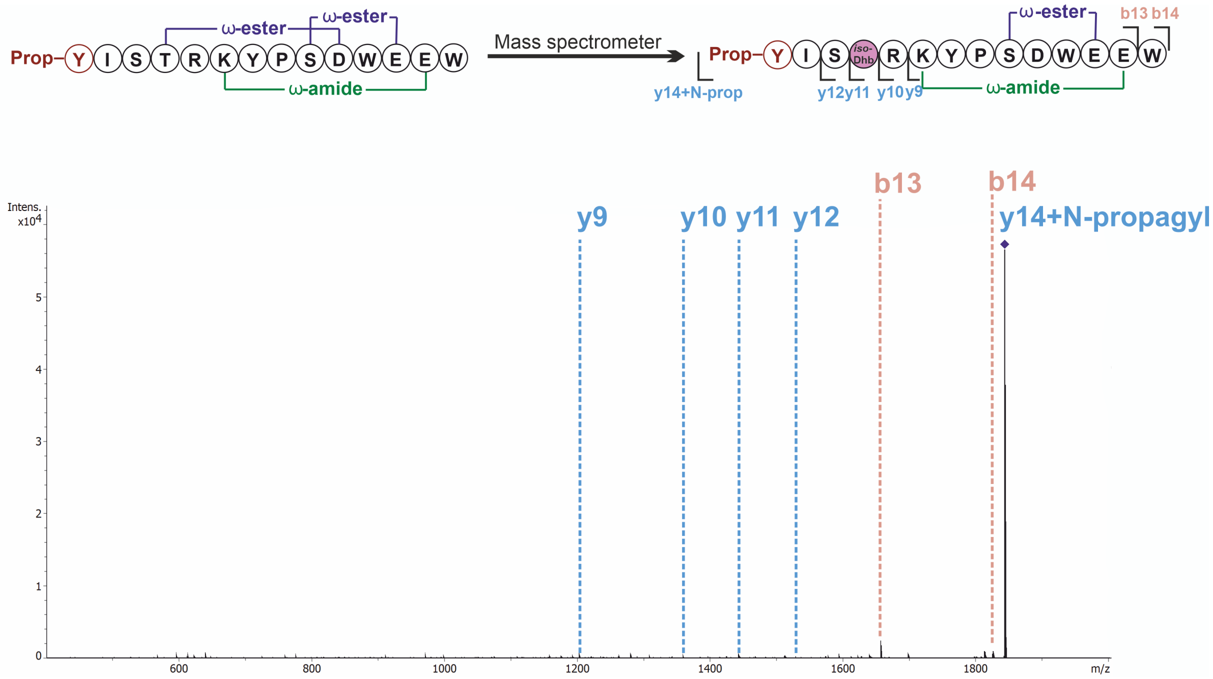

Figure S33. MALDI-TOF MS/MS spectrum of tricyclic MvJ_CP_N(Prop) (microviridin J core peptide with N-terminal *O*-propargyl-L-tyrosine) (**3**) , which shows a fragmentation pattern diagnostic for the tricyclic architecture of microviridins. As previously reported,[1] the N-terminal lactone ring of microviridin opens during MALDI-TOF MS/MS analysis due to a rearrangement reaction, yielding a dehydrated threonine (*iso*-dehydrobutyrine; *iso*-Dhb) and the carboxylic acid moiety of aspartate instead of a lactone bond (Fig. S37). For a comprehensive list of all calculated and observed fragment ions see the supplementary Excel file.

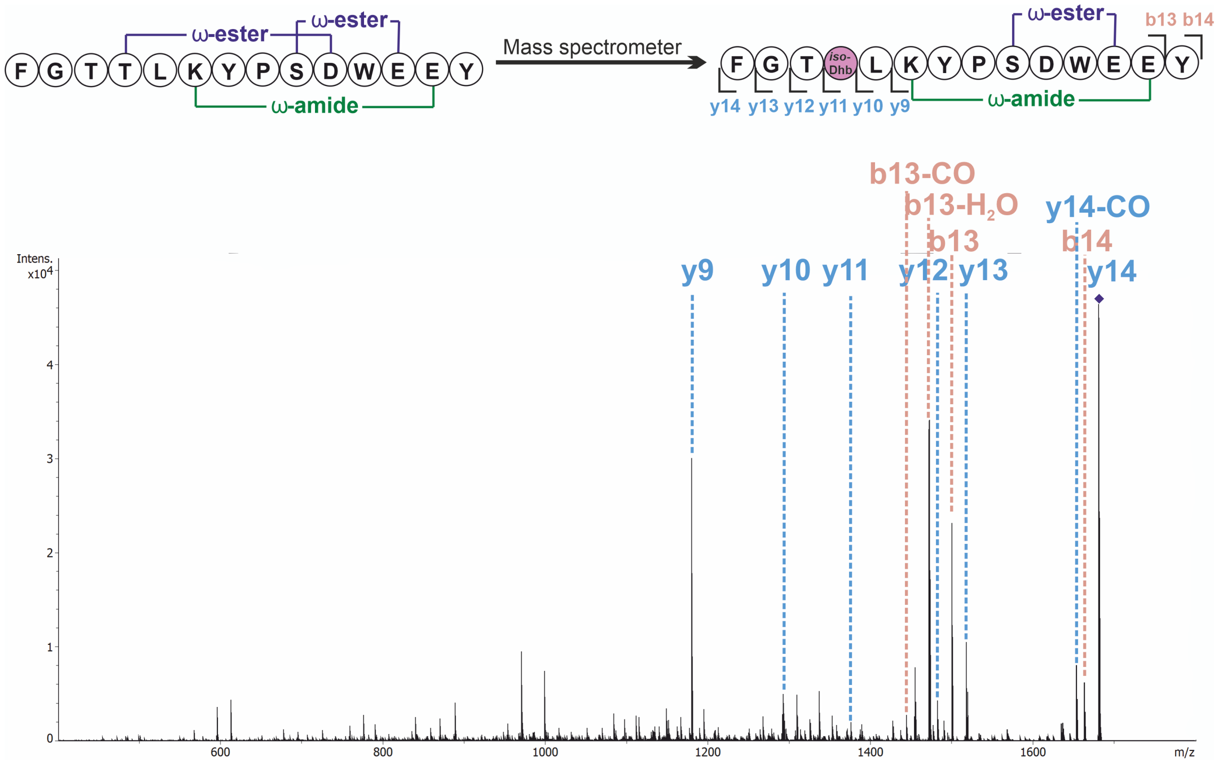

Figure S34. MALDI-TOF MS/MS spectrum of tricyclic MvB_CP (microviridin B core peptide) (**4**), which shows a fragmentation pattern diagnostic for the tricyclic architecture of microviridins. As previously reported,[1] the N-terminal lactone ring of microviridin opens during MALDI-TOF MS/MS analysis due to a rearrangement reaction, yielding a dehydrated threonine (*iso*-dehydrobutyrine; *iso*-Dhb) and the carboxylic acid moiety of aspartate instead of a lactone bond (Fig. S37). For a comprehensive list of all calculated and observed fragment ions see the supplementary Excel file.

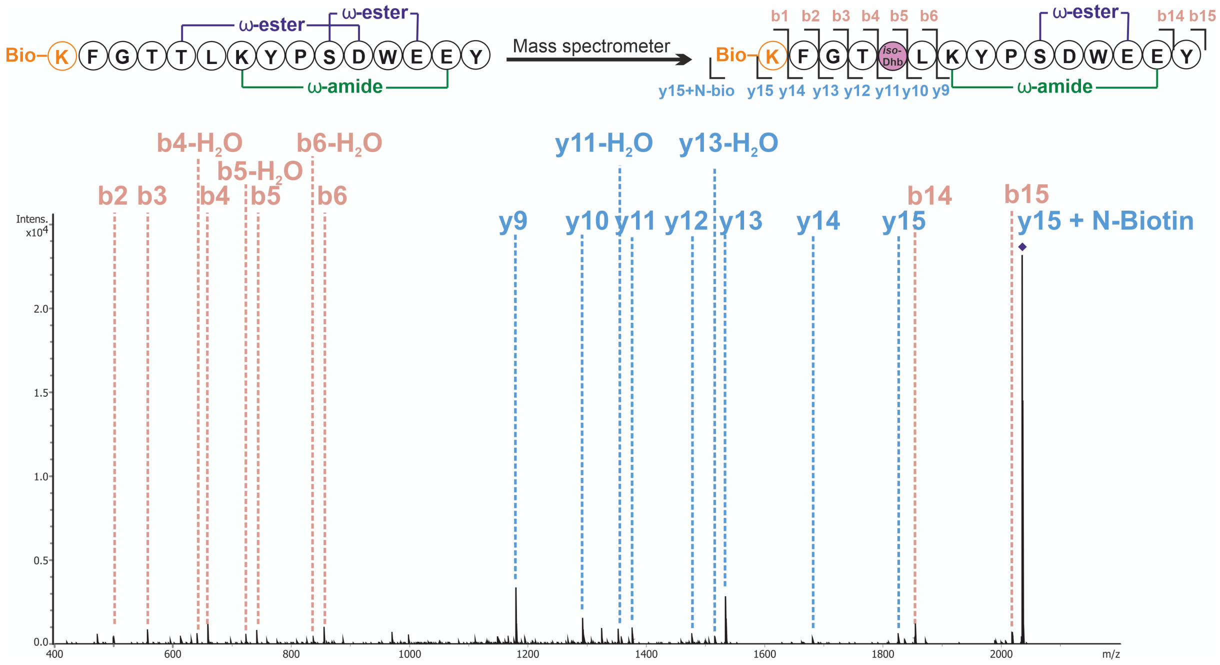

Figure S35. MALDI-TOF MS/MS spectrum of tricyclic MvB_CP_N(Bio) (microviridin B core peptide with N-terminal N^{ε}-biotinyl-L-lysine) (**5**), which shows a fragmentation pattern diagnostic for the tricyclic architecture of microviridins. As previously reported,[1] the N-terminal lactone ring of microviridin opens during MALDI-TOF MS/MS analysis due to a rearrangement reaction, yielding a dehydrated threonine (*iso*-dehydrobutyrine; *iso*-Dhb) and the carboxylic acid moiety of aspartate instead of a lactone bond (Fig. S37). For a comprehensive list of all calculated and observed fragment ions see the supplementary Excel file.

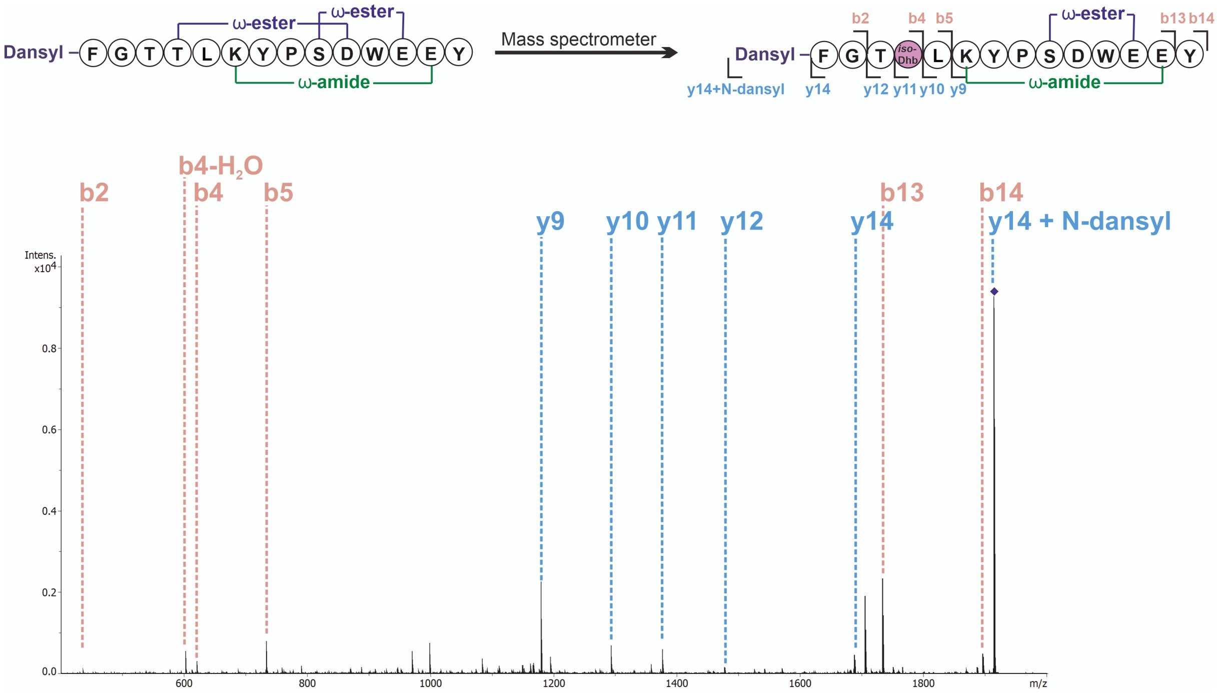

Figure S36. MALDI-TOF MS/MS spectrum of tricyclic MvB_CP_N(dansyl) (microviridin B core peptide with a N-terminal dansyl group) (**6**), which shows a fragmentation pattern diagnostic for the tricyclic architecture of microviridins. As previously reported,[1] the N-terminal lactone ring of microviridin opens during MALDI-TOF MS/MS analysis due to a rearrangement reaction, yielding a dehydrated threonine (*iso*-dehydrobutyrine; *iso*-Dhb) and the carboxylic acid moiety of aspartate instead of a lactone bond (Fig. S37). For a comprehensive list of all calculated and observed fragment ions see the supplementary Excel file.

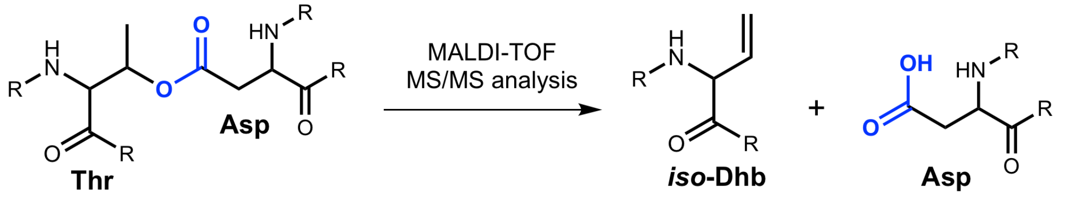

Figure S37. Ring-opening reactions during MS/MS analysis. Opening of the N-terminal lactone ring of microviridin due to a rearrangement reaction yielding a dehydrated threonine (*iso*-dehydrobutyrine; *iso*-Dhb) and the carboxylic acid moiety of aspartate. This reaction is frequently observed in MALDI-TOF MS/MS analysis of microviridins and microviridin-like compounds.[1]

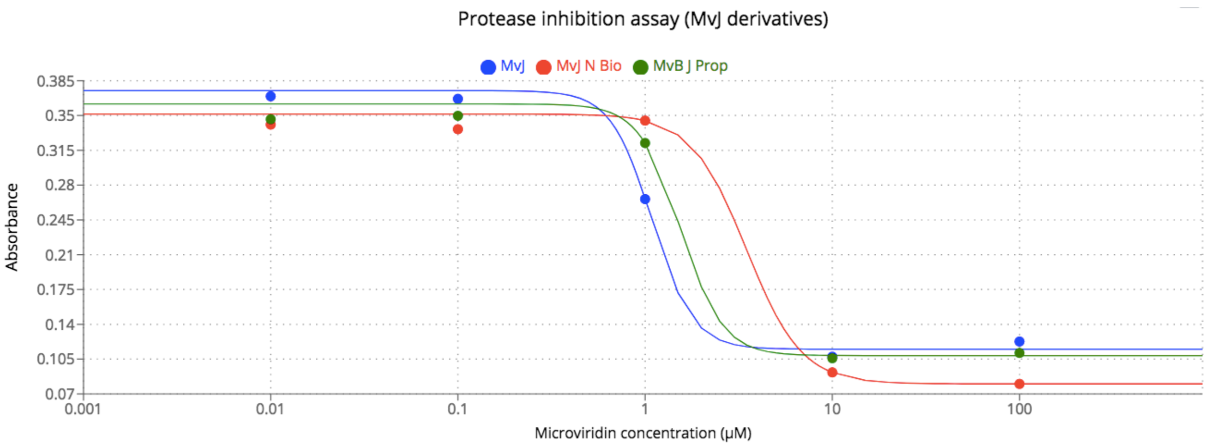

Figure S38. Results of the protease inhibition assay of the microviridin J derivatives. The plots were created with the Quest Graph™ IC_{50} Calculator.[2]

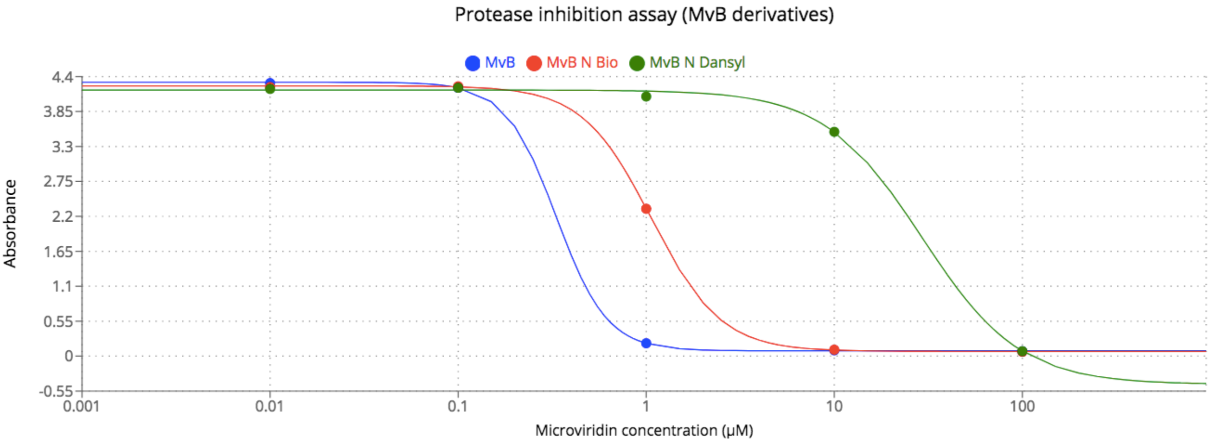

Figure S39. Results of the protease inhibition assay of the microviridin B derivatives. The plots were created with the Quest Graph™ IC50 Calculator.[2]

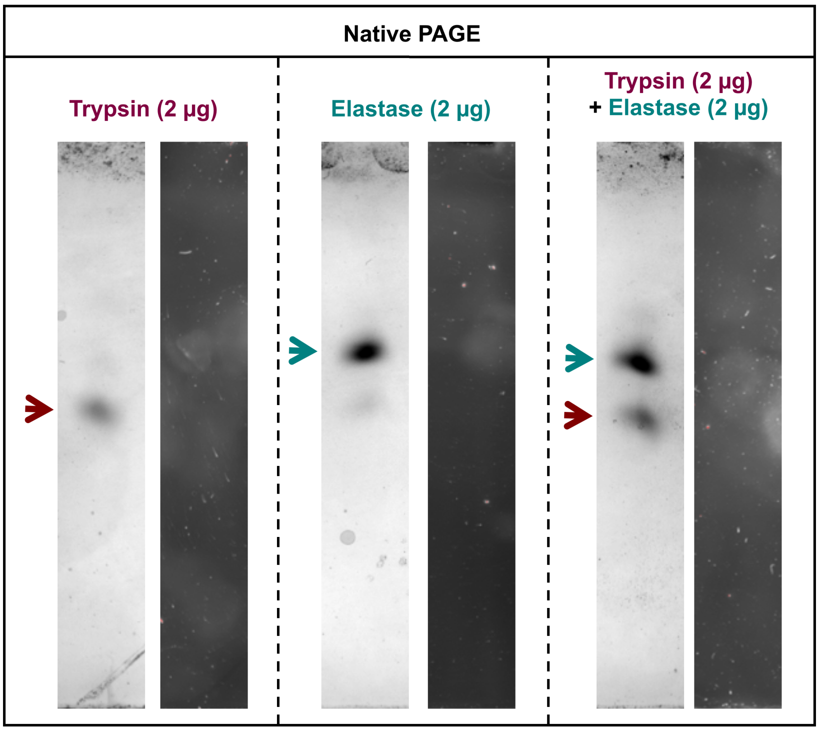

Figure S40. Blank control for protease labeling assay.

References

[1] a) D. Dehm, J. Krumbholz, M. Baunach, V. Wiebach, K. Hinrichs, A. Guljamow, T. Tabuchi, H. Jenke-Kodama, R. D. Süssmuth, E. Dittmann, *ACS Chem. Biol.* **2019**, *14*, 1271-1279; b) H. Lee, Y. Park, S. Kim, *Biochemistry* **2017**, *56*, 4927-4930; c) H. Roh, Y. Han, H. Lee, S. Kim, *Chembiochem* **2019**, *20*, 1051-1059.

[2] "Quest Graph™ IC50 Calculator." AAT Bioquest, Inc., 18 May. 2022, https://http://www.aatbio.com/tools/ic50-calculator.
